# Monitoring Receptor Clustering by Aggregation‐Induced Emission

**DOI:** 10.1002/cplu.202500286

**Published:** 2025-09-03

**Authors:** Robert Bekus, Kevin Rudolph, Steffen Riebe, Jens Voskuhl, Thomas Schrader

**Affiliations:** ^1^ Faculty of Chemistry University of Duisburg‐Essen Universitätsstraße 7 45117 Essen Germany

**Keywords:** aggregation‐induced emission, amphiphiles, lipid bilayer, receptor clustering, signal transduction, supramolecular chemistry

## Abstract

This study introduces a simple signal transduction system that mimics the receptor tyrosine kinase mechanism by employing amphiphilic receptors embedded in lipid bilayers. The designed receptors carry bisphosphonate head groups and feature aggregation‐induced emission enhancement (AIEE) properties. Upon addition of polyammonium messengers, they undergo ligand‐induced dimerization or clustering inside the membrane. Steric restriction of intramolecular motion in the AIE luminophores sends out a fluorescence signal. Systematic comparative studies highlight the impact of receptor design, lipid environment and messenger properties on the efficiency, kinetics, and strength of signal transduction. These findings provide new insight into the interplay between receptor aggregation and membrane organization in controlling fluorescence‐based signaling systems. Practical perspectives and inherent limitations are critically discussed.

## Graphical abstract

Receptor clustering induced by molecular recognition: AIE‐functionalized bisphosphonate receptors are embedded in a lipid bilayer. Upon addition of a cationic messenger, they undergo dimerization and send out a fluorescence signal.

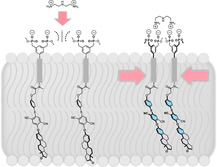

## 1 Introduction

Signal transduction across lipid membranes is a cornerstone of cellular communication, in some cases mediated by receptor clustering and dimerization.^[^ ^1^ ^]^ This process plays a vital role in amplifying extracellular signals and converting them into precise intracellular responses. Highly specified transmembrane proteins are stimulated by small molecule primary messengers, membrane potential or light. The incoming information is then transferred into the cell interior by means of releasing a secondary messenger and subsequent amplification in a catalytic reaction cascade.

Surprisingly, until recently, chemists did not attempt to imitate this fundamental natural process with artificial examples. In the past two decades, however, an increasing number of groups have explored synthetic signaling systems, often utilizing a built‐in fluorescent readout.^[^ ^2^ ^]^

Early work focused on imitating natural examples while more recently, new principles were introduced in artificial signaling, which are not found in Nature. Most signaling proteins follow one of two mechanistic paths for signal transmission across the bilayer: either docking of the incoming messenger leads to a major conformational change inside the transmembrane units of the receptor (GPCRs = G‐protein coupled receptors), or two membrane‐spanning separate protein modules are bridged by the primary messenger and dimerize (RTKs = receptor tyrosine kinases). The RTK principle inspired chemists and was imitated with steroid‐based TM building blocks carrying designed head and tail elements (Hunter et al.).^[^ ^3^ ^,^ ^4^ ^]^ A true molecular recognition event was built‐in by the Schrader group attaching bisphosphonate head groups to the TM building blocks, which were dimerized by externally added polyammonium messengers.^[^ ^5^ ^,^ ^6^ ^]^ Recently a different DNA‐based scaffold was introduced by Liu et al.^[^ ^7^ ^]^

On the contrary, the GPCR principle has also spurred research on externally triggered conformational changes inside lipid bilayers (Clayden et al.): thus peptide foldamers with attached azobenzene chromophore were switched with visible light between a left‐ and right‐handed conformational state.^[^ ^8^ ^]^ Later the foldamer was equipped with a carboxylate binding site, and complexation of a chiral carboxylate messenger on one end was translated into a turned‐on fluorescence of a pyrene excimer at the other end.^[^ ^9^ ^]^

In the attempt to address the disturbing background signal inherently present in most of the bioinspired signal transduction systems, new artificial transmission mechanisms were explored, pioneered by the Hunter group. A highly successful approach is the construction of a short membrane‐embedded functional shuttle, which travels back and forth between the membrane exterior and interior, activated by an incoming signal and itself starting a catalytic cascade which sends out the amplified signal. To this end, the signal transducer carries a highly polar head group, which can be turned into an unpolar moiety by interaction with the incoming messenger (pH,^[^ ^10^ ^]^ metal complexation,^[^ ^11^ ^]^ and redox switching.^[^ ^12^ ^]^ The catalyzed reaction releases a fluorescent reporter,^[^ ^10^ ^]^ or a co‐encapsulated dye.^[^ ^13^ ^]^ Light‐controlled signal transduction was realized in related systems.^[^ ^14^ ^,^ ^15^ ^]^

In an elegant new development, the Langton group very recently presented an intervesicle signaling system, which relies on the double function of clioquinol as a diffusable signal and cationophore.^[^ ^16^ ^]^ Finally, the Zelikin group created another highly efficient signal transduction mechanism employing self‐immolative linkers.^[^ ^17^ ^]^

In all above artificial signal transduction systems, one important aspect appears underrepresented, that is, a direct proof of receptor clustering, a very important principle in cell communication. Inspired by the simplicity of the receptor tyrosine kinase,^[^ ^18^ ^]^ principle, we have now developed a new synthetic approach using amphiphilic receptors with aggregation‐induced emission (AIE) properties to explore fluorescence‐based signal transduction. In such a system receptor clustering is directly translated into induced fluorescence emission and only one single transmembrane receptor is required.

The term AIE was coined nearly 25 years ago,^[^ ^19^ ^]^ when Tang et al. discovered that simple siloles containing phenyl‐rotors exhibit bright emission only when aggregated or in the solid state.^[^ ^20^ ^]^ Although this phenomenon has been observed almost 150 years ago by Schmidt et al.,^[^ ^21^ ^]^ this unique feature of highly flexible luminophores has experienced a renaissance in numerous application areas ranging from biomedical chemistry^[^ ^22^ ^]^ to materials sciences.^[^ ^23^ ^]^

We chose the combination of transmembrane receptors with an AIE readout because of the high sensitivity and precision of fluorescence signals, which allow for real‐time monitoring of receptor clustering and ligand interactions in complex lipid environments. Such systems leverage the unique photophysical properties of AIE luminophores, which emit fluorescence only when molecular motion is restricted, such as during aggregation.^[^ ^24^ ^]^ This behavior has been extensively utilized in various biosensing applications,^[^ ^25^ ^]^ where aggregation‐induced emission serves as a reliable readout for molecular interactions in complex environments (e.g., lipid bilayers).^[^ ^26^ ^]^

To study the factors governing signal efficiency, we focused on two similar receptor designs, which exhibit three key components: A bisphosphonate‐functionalized recognition unit, a steroidal hydrophobic spacer and a terminal AIE luminophore. The bisphosphonate group was chosen for its high affinity to polyammonium messengers, enabling robust signal induction even in a competitive aqueous environment.^[^ ^27^ ^]^ It carries the membrane‐anchor of both receptors, a lithocholic acid derivative, which in turn is linked via a dialkyne unit or a 1,2,3‐triazole to the terminal AIE group. Both linker groups, the dialkyne and the 1,2,3‐triazole promote rigidity and linearity within the receptor structure. This design ensures the formation of an extended membrane‐spanning conformation and produces messenger‐induced receptor aggregates by aligning two or more receptor molecules inside the membrane. If receptor docking brings large unpolar surfaces of the aligned receptors into close proximity, powerful van‐der‐Waals interactions will lead to restriction of intramolecular motion (RIM),^[^ ^28^ ^]^ ultimately amplifying the fluorescence signal (AIE).

**Figure 1:**
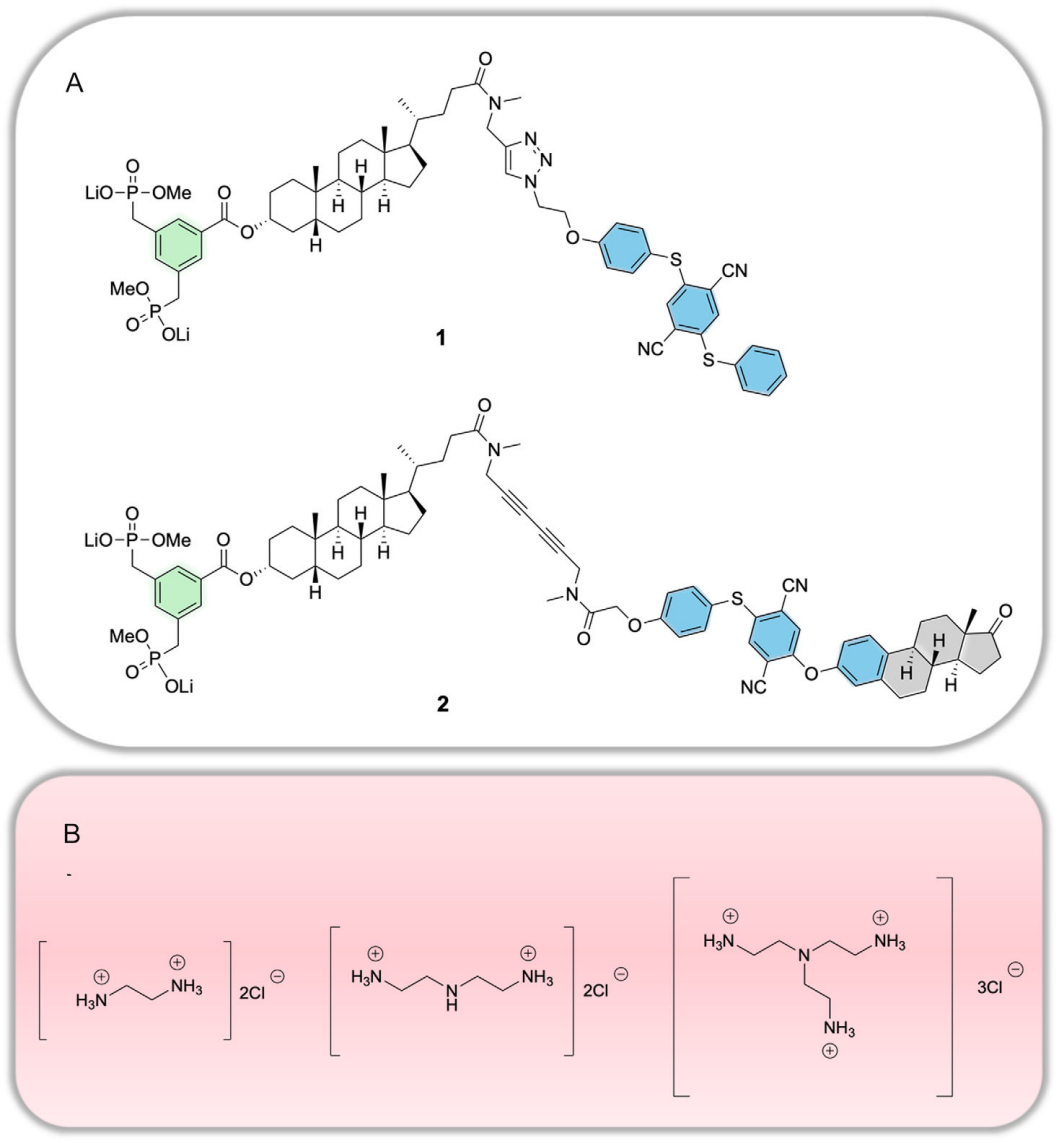
A) Molecular structure of the amphiphilic receptor molecules **1** and **2**. Both carry a bisphosphonate dianion (green) for messenger recognition, *a central steroidal linker* and an AIE luminophore core (blue) for signal emission. Receptor **2** is further elongated with an attached estrone (gray). B) Structures of polyamines. From left to right: H_2_EDA^2+^, H_2_DET^2+^ and H_3_TREN^3+^.

Both AIE receptors **1** and **2** are depicted in **Figure** **1**A. They share a similar AIE luminophore core structure, based on aromatic ethers (highlighted in blue), which endow them with luminescent properties.^[^ ^29^ ^]^ A key distinction lies in the extended steroid backbone of estrone (highlighted in gray) in compound **2** which enlarges its overall unpolar surface and thus strengthens its intermolecular interactions and boosts receptor clustering. The structural similarity between the estrone unit and cholesterol is hypothesized to enhance the integration of receptor **2** into heterogeneous model membranes, which contain cholesterol as integral lipid component. Furthermore, the estrone‐based receptor will readily self‐aggregate in a signal transduction process, and form lipid rafts, which in turn amplify the incoming signal from the primary messenger.

Both receptors **1** and **2** were embedded two different lipid environments, that is, in homogeneous (DOPC) and phase‐separated (DOPC/DPPC/Chol)^[^ ^30^ ^]^ lipid bilayers. Their responses to various externally added ionizable polyamine messengers (Figure 1B), such as diprotonated ethylenediamine (H_2_EDA^2+^), diprotonated diethylenetriamine (H_2_DET^2+^), and triprotonated tris(2‐aminoethyl)amine (H_3_TREN^3+^), were systematically investigated. The overarching goal was to understand how molecular design and membrane environment influence signaling efficiency and receptor clustering.

### 1.1 Concept of the Signal Transduction System with AIE‐Functionalized Receptors

**Figure 2:**
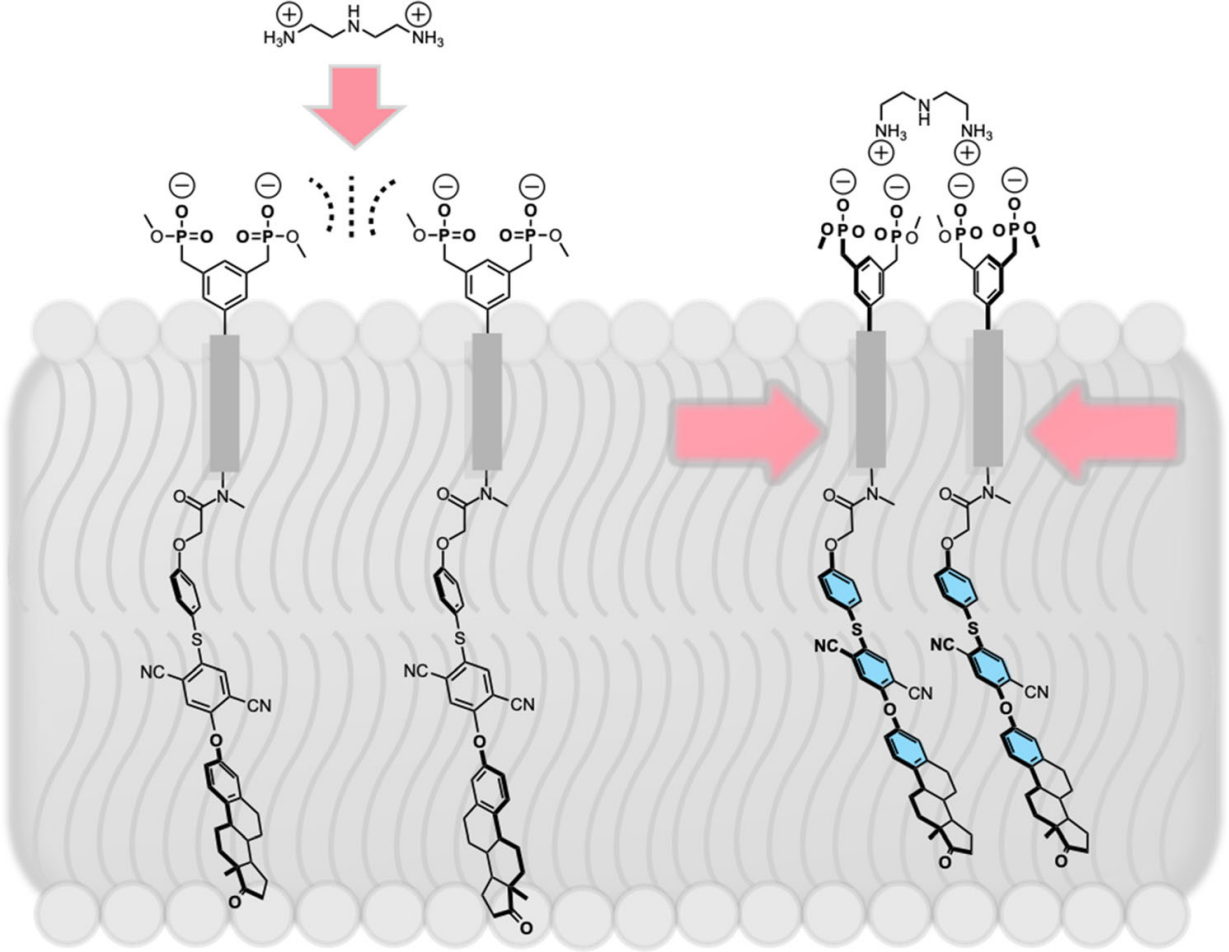
Schematic representation of the signal transduction system with AIE‐functionalized bisphosphonate receptors embedded in a lipid bilayer. Due to electrostatic repulsion of their anionic head groups, the two receptor units are separated, remaining in a non‐aggregated state (left). External addition of the dicationic messenger (e.g., H_2_DET^2+^) leads to powerful complex formation and bridges them into dimers (right), resulting in aggregation‐induced emission enhancement (AIEE).

**Figure** **2** illustrates the newly developed signal transduction model, featuring AIE‐functionalized receptors embedded in a lipid bilayer. Receptors **1** and **2** are shown in their initial non‐aggregated states. Assuming an extended conformation, the head‐to‐tail distance of the receptors is ≈40 Å (**1**) and ≈47 Å (**2**), which is within the typical bilayer thickness range of 40–50 Å observed in DOPC^[^ ^31^ ^]^ and DOPC/Chol^[^ ^32^ ^]^ liposomes. These dimensions indicate that the receptors’ length aligns closely with the thickness of the lipid bilayer, a prerequisite for membrane spanning.

In the AIE‐based system, amphiphilic receptors exist as monomers in close proximity within the lipid bilayer. Repulsive Coulomb interactions between the bisphosphonate groups prevent spontaneous clustering, ensuring that no unintended fluorescence signal is generated. Upon the addition of a polyammonium‐based messenger (e.g., H_2_DET^2+^), at least two receptors are bridged extracellularly via a chelate–like complex. This ligand‐induced complexation stabilizes receptor dimers through hydrogen bonding, cation‐*π* interactions, and attractive Coulomb forces. The resulting proximity of the receptors induces clustering of the AIE luminophores, leading to aggregation‐induced emission enhancement (AIEE) due to motional hindrance, which is detected as a fluorescence signal.

Unlike systems that allow for heterodimeric complex formation, the AIE receptors are exclusively homodimeric because only one type of receptor is incorporated into the liposomes. This ensures that all ligand‐induced dimers contribute effectively to fluorescence enhancement.

Due to the transversal alignment of the AIE receptors within the bilayer, intermolecular interactions that disrupt the original lipid packing^[^ ^33^ ^]^ density in the hydrophobic region of the liposome may promote receptor clustering. These receptor aggregates are hypothesized to accumulate within lipid domains in phase‐separated membrane systems, such as those found in DOPC/DPPC/Chol liposomes. Within these domains, the bisphosphonate groups of the receptors statistically orient equally toward the external aqueous environment (as shown in Figure 2), but also towards the internal aqueous compartment (unproductive for signaling), depending on their positioning in the bilayer.

The AIE luminophore of the receptors extends from the hydrophobic membrane core, which has a polarity similar to hexane, toward the interface with the internal aqueous medium. This orientation is driven by the luminophore's polarity, which positions it near the hydrophilic region of the bilayer. Upon complexation of two or more independent bisphosphonate groups on the external side of the bilayer with a bridging polyammonium ligand, the AIEE effect is triggered, resulting in a measurable fluorescence signal. This phenomenon couples tight complex formation with efficient restriction of the rotational motion of the luminophore within the membrane, amplifying its AIE properties.

The liposomes used in the system were doped with a single type of receptor (**1** or **2**), leading to the formation of homodimeric or homotrimeric receptor‐ligand complexes upon interaction with external messengers. These structural arrangements are believed to play a crucial role in enhancing the efficiency and precision of the signal transduction process.

The structural design of the AIE receptors ensures compatibility with the lipid bilayer, while their ability to form aggregates allows them to exploit the dynamic nature of the membrane. The receptors’ orientation and organization within the bilayer are influenced by the lipid composition and phase behavior of the membrane, with DOPC/DPPC/Chol membranes providing a model for phase‐separated systems. These environmental factors not only affect the receptors’ conformational states but also modulate their interaction with external messengers.

The system is optimized for controlled fluorescence responses, with bisphosphonate–ligand interactions leading to immediate and detectable changes in fluorescence intensity. The modular nature of the receptors allows (in principle) for systematic adjustments to their structure and functionality, making them highly adaptable for a range of experimental conditions and signaling applications.

While the concept of signal transduction using AIE‐functionalized receptors is fundamentally robust, certain strategies from adjacent lipid‐modification methodologies could further optimize receptor orientation and functional output. Techniques such as post‐insertion of modified lipid components, commonly used to enhance the surface properties of liposomes (e.g., PEGylation), might be adapted to promote a more outward‐facing orientation of receptor headgroups.^[^ ^34^ ^]^

However, preliminary experiments applying similar strategies to the AIE system resulted in suboptimal fluorescence output. This suggests that while such methods hold potential for improving receptor alignment, their impact on fluorescence efficiency must be critically assessed. These findings underscore the delicate balance between receptor orientation, aggregation efficiency, and the resulting signal transduction dynamics.

## 2 Results and Discussion

### 2.1 Synthesis of Messengers and Receptors

To investigate artificial signal transduction, three compact polyamines—EDA (ethylenediamine), DET (diethylenetriamine), and TREN (tris(2‐aminoethyl)amine)—were utilized as messengers. These polyamines were converted into their hydrochloride salts by reacting their ethanolic solutions with concentrated hydrochloric acid (37% HCl). The resulting ammonium salts were precipitated, isolated as solids, and dried. This step was performed primarily for purification and stabilization of the polyamines, as amino groups are prone to oxidation. Aqueous solutions (pH 7.4) of these polyammonium salts were prepared using deionized water for the experiments.

Due to the pKa values of secondary (pKa 4.42) and tertiary (pKa 2.60 in KNO_3_ solution, 1.57 in NaCl solution) amines, DET likely carries two positive charges, while TREN carries three positive charges at pH 7.4. These partial protonation states result from unfavorable intramolecular repulsions between protonated NH^+^ groups, caused by the short C_2_ spacers. The chemical structures and net charges of EDA, DET, and TREN are depicted in Figure 2.

**Figure 3:**
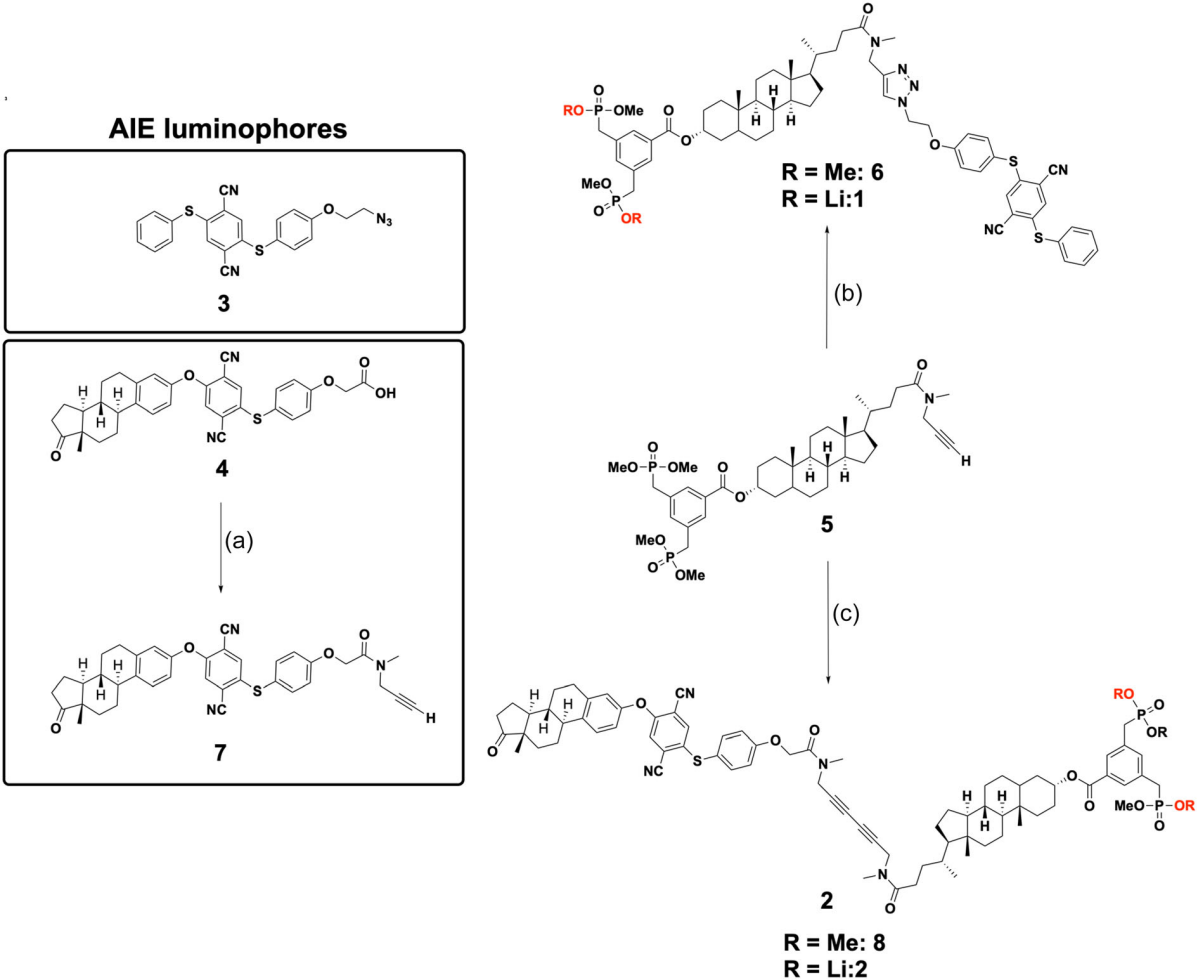
Synthesis scheme of AIE amide **7** and AIE receptors **1** and **2**: a) *Amidation of AIE luminophore*: EDC·HCl, 4‐DMAP, DCM, 68%. b) *Click Reaction*: Cu(CNMe_4_)PF_6_, TBTA, DCM, rt, overnight, 75%; LiI‐mediated demethylation: LiI, THF, reflux, overnight, quantitative. c) *Glaser‐Hay Coupling*: CuCl, DCM, TMEDA, O_2_, rt, 5 h, 52%; LiI‐mediated demethylation: LiI, THF, reflux, overnight, quantitative.

The new transmembrane receptors consist of AIE luminophores (**3** or **4** ^**[**^ ^35^ ^]^) and a lipid anchor derived from lithocholic acid (**5** ^[^ ^36^ ^]^). The AIE components, carrying either a terminal azide or a carboxylic acid, were conjugated to the TM intermediate **5** via distinct synthetic pathways depicted in **Figure** **3**.

AIE receptor **1** was synthesized by means of a copper(I)‐catalyzed azide–alkyne click reaction. To this end, TM intermediate **5** was coupled with the AIE luminophore **3** in the presence of tris[(1‐benzyl‐1H‐1,2,3‐triazol‐4‐yl)methyl]amine (TBTA) in dichloromethane (DCM) at room temperature. The resulting AIE‐functionalized receptor precursor **6** (75%) was subsequently treated with lithium iodide (LiI) in anhydrous tetrahydrofuran (THF) to afford lithium salt **1** in quantitative yield.

To enable receptor **2** construction, estrone luminophore **4** was functionalized with *N*‐methylpropargylamine via EDC‐mediated amidation, yielding estrone‐propargylamide **7** as an *E/Z* isomeric mixture (68%). Subsequent Glaser‐Hay coupling of **7** with TM intermediate **5** under oxygen with catalytic copper(I) chloride and TMEDA gave receptor precursor **8** (52%). Quantitative dealkylation with LiI furnished receptor **2** as lithium salt.

### 2.2 Investigation of Signal Transmission in a Homogeneous DOPC Lipid Membrane

As a starting point, experiments were conducted in a homogeneous lipid membrane environment composed solely of DOPC. This model represents a simplified system compared to more complex heterogeneous lipid mixtures. Liposomes (large unilamellar vesicles, LUVs) with a diameter of 200 nm were prepared by co‐incorporating DOPC and AIE‐functionalized receptors (**1** or **2**) to achieve a total lipid–receptor concentration of 2 mM, with 5 mol% of the total composition corresponding to the receptor. The lipid–receptor mixture, considered as a single amphiphilic system, was hydrated and suspended in demineralized water at pH 7.4. This approach avoided interference from buffer systems, which universally contain ionic species. Such ions can shield or disrupt the electrostatic interactions between the polycationic messengers (H_3_TREN^3+^, H_2_DET^2+^, and H_2_EDA^2+^) and the negatively charged bisphosphonate groups on the receptors. By using demineralized water, these competing electrostatic interactions were minimized, ensuring that receptor–ligand interactions and receptor clustering remained unaffected by external ionic influences. This approach maintained the native electrostatic environment, allowing a clearer observation of the role of polycationic messengers in signal transmission.

A receptor concentration of 5 mol% was identified as optimal for efficient signal transduction. While 2.5 mol% led to detectable fluorescence enhancement, higher receptor densities further amplified the signal. However, at 10 mol%, no additional fluorescence increase was observed despite the doubled receptor concentration, indicating a saturation effect. Additional data are provided in the Supporting Information (Figure S7–9).

**Figure 4:**
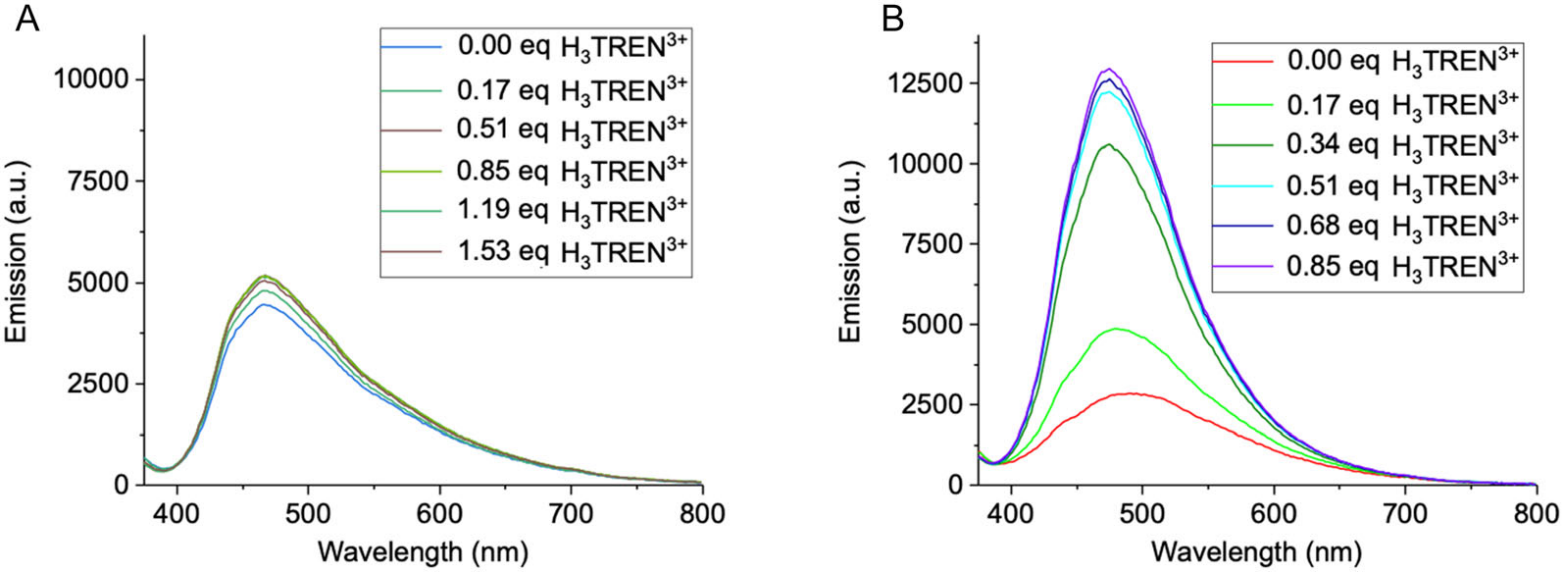
Fluorescence response of receptors **1** A) and **2** B) upon titration with a higher concentration of H_3_TREN^3+^ (*V* = 1.1 μL, *c *= 0.93 mM, 0.17 eq) in deionized water at pH 7.4. In this system, receptor **2** (spectrum B) exhibited a ≈4.6‐fold fluorescence increase (≈0.5 eq H_3_TREN^3+^), demonstrating that further fluorescence enhancement could be induced by higher ligand levels. In contrast, receptor **1** (spectrum A) showed no significant signal transduction, even at ≈1.5 eq. The titration was performed until fluorescence emission reached a stable level, with no further detectable changes. The observed fluorescence response indicates that the additional estrone moiety in **2** promotes efficient receptor clustering even in a homogeneous DOPC environment.

The addition of the well‐characterized polycationic messengers H_3_TREN^3+^, H_2_DET^2+^, and H_2_EDA^2+^ to DOPC liposome suspensions containing receptor **1** did not induce a significant AIEE effect (**Figure** **4**A, see also Figure S13B,C, Supporting Information for H_2_DET^2+^and H_2_EDA^2+^). This is surprising because very similar TM units have earlier been employed by us and led to powerful signal transduction.^[^ ^5^ ^]^ Most likely the homogeneous membrane environment alone does not sufficiently support receptor clustering and signal transmission.

By contrast efficient AIE signal amplification was observed for receptor **2**. Upon incremental addition of 0.17 equivalent aliquots of H_3_TREN^3+^ (0.5 equivalents in total), a 4.6‐fold increase in fluorescence intensity was achieved, with a nearly instantaneous response to messenger addition, emphasizing the system's high sensitivity to ligand binding (Figure 4B). This immediate activation was particularly pronounced for H_3_TREN^3+^, which exhibited a strong response even at lower concentrations, whereas H_2_DET^2+^ required higher amounts to achieve similar effects (Supporting Information, Figure S13E). While fluorescence enhancement again occurred almost immediately upon messenger addition, it only began after successive additions of 0.25 H_2_DET^2+^, suggesting a threshold concentration was necessary to initiate receptor clustering. Finally, H_2_EDA^2+^ produced the weakest effect of all messengers (Figure S13F, Supporting Information).

We conclude that highly charged polyammonium messengers undergo a superior receptor–messenger interaction and show a greater potential for receptor clustering, indicated by stronger signal amplification.

It should be noted that the saturation of AIE receptors with messengers generally exceeds the theoretical stoichiometric ratios of 3:1 for H_3_TREN^3+^ and 2:1 for H_2_DET^2+^ and H_2_EDA^2+^. Due to a loss of 50% of unproductive receptor orientations with receptor heads pointing into the liposome, the maximum induced fluorescence signal would be expected at  ≈0.17 equivalents of H_3_TREN^3+^ and 0.25 equivalents of H_2_DET^2+^and H_2_EDA^2+^. The reasons for the higher degree of saturation remains unclear at this moment.

A surprising feature of the new AIE signaling system is the instantaneous appearance of the fluorescence signal after messenger addition, which did not change over time. The signal transduction times were so short, that conventional kinetic measurements could not be performed. This means that signal transduction occurred within seconds. In sharp contrast, signal transduction within the same system (identical transmembrane units and host guest recognition) took 10–15 min until it reached the maximum effect, if a chemical reaction was combined with receptor dimerization.^[^ ^6^ ^]^

Figure S16, Supporting Information depicts the rapid emission increase and its stability over 15 min as column pairs of a typical titration experiment.

**Figure 5:**
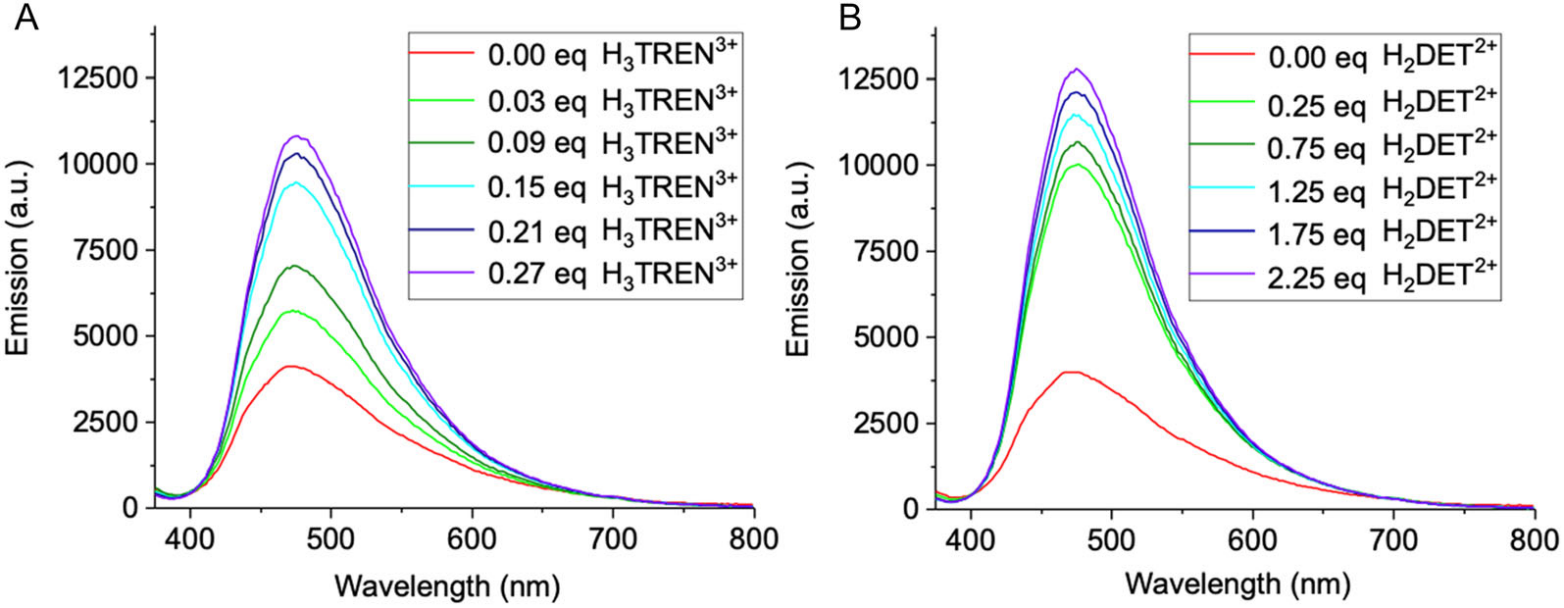
Signal transduction experiments in phase‐separated LUVs composed of the lipids DOPC/DPPC/Chol (1:2:1, *c *= 2 mM). The liposomes were doped with 5 mol% (0.1 mM) of receptor **1** and suspended in ultrapure water (pH 7.4, adjusted with NaOH) to minimize interference from competing ions (e.g., from buffer components) while maintaining physiological pH conditions. Each experiment was performed by titrating a 60 μL sample with an external signaling molecule until fluorescence emission reached a stable level. The titration was performed using aqueous solutions of H_3_TREN^3+^ (*V *= 2.15 μL, *c *= 93 μM, 0.03 eq) and H_2_DET^2+^ (*V* = 1 μL, *c *= 1.5 mM, 0.25 eq). The titration experiments were conducted at 25 °C. In the absence of interfering ions, H_3_TREN^3+^ and H_2_DET^2+^ both elicited a strong signal already at substoichiometric amounts, with maximum signal enhancements of 2.6‐fold A) and 3.2‐fold B). The induced fluorescence signals remained stable throughout and beyond the titration, demonstrating reproducibility.

**Figure 6:**
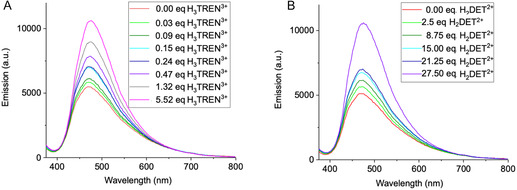
Signal transduction experiments in phase‐separated LUVs composed of the lipids DOPC/DPPC/Chol (1:2:1, *c *= 2 mM). The liposomes were doped with 5 mol% (0.1 mM) of receptor **1** and were suspended as an aqueous vesicle dispersion in HEPES buffer (100 mM, pH 7.4). Each experiment was performed by titrating a 60 μL sample with an external signaling molecule until fluorescence emission reached a stable level. The titration was performed using aqueous solutions of H_3_TREN^3+^ (1. *V* = 2.15 μL, *c *= 93 μM, 0.03 eq; 2. *V* = 1.08 μL, *c *= 0.93 mM, 0.17 eq; *V* = 2.7 μL, *c *= 9.3 mM, 4.2 eq) and H_2_DET^2+^ (1. *V* = 1 μL, *c *= 1.5 mM, 0.25 eq; 2. *V *= 2.48 μL, *c *= 15.1 mM, 6.2 eq). The titration experiments were conducted at 25 °C. In the presence of interfering ions, H_3_TREN^3+^ stands out as most efficient messenger, requiring much smaller amounts than H_2_DET^2+^ for the same emission increase, and exhibiting rapid response. Maximum signal enhancements reached 1.9‐fold A) and 2.1‐fold B).

**Figure 7:**
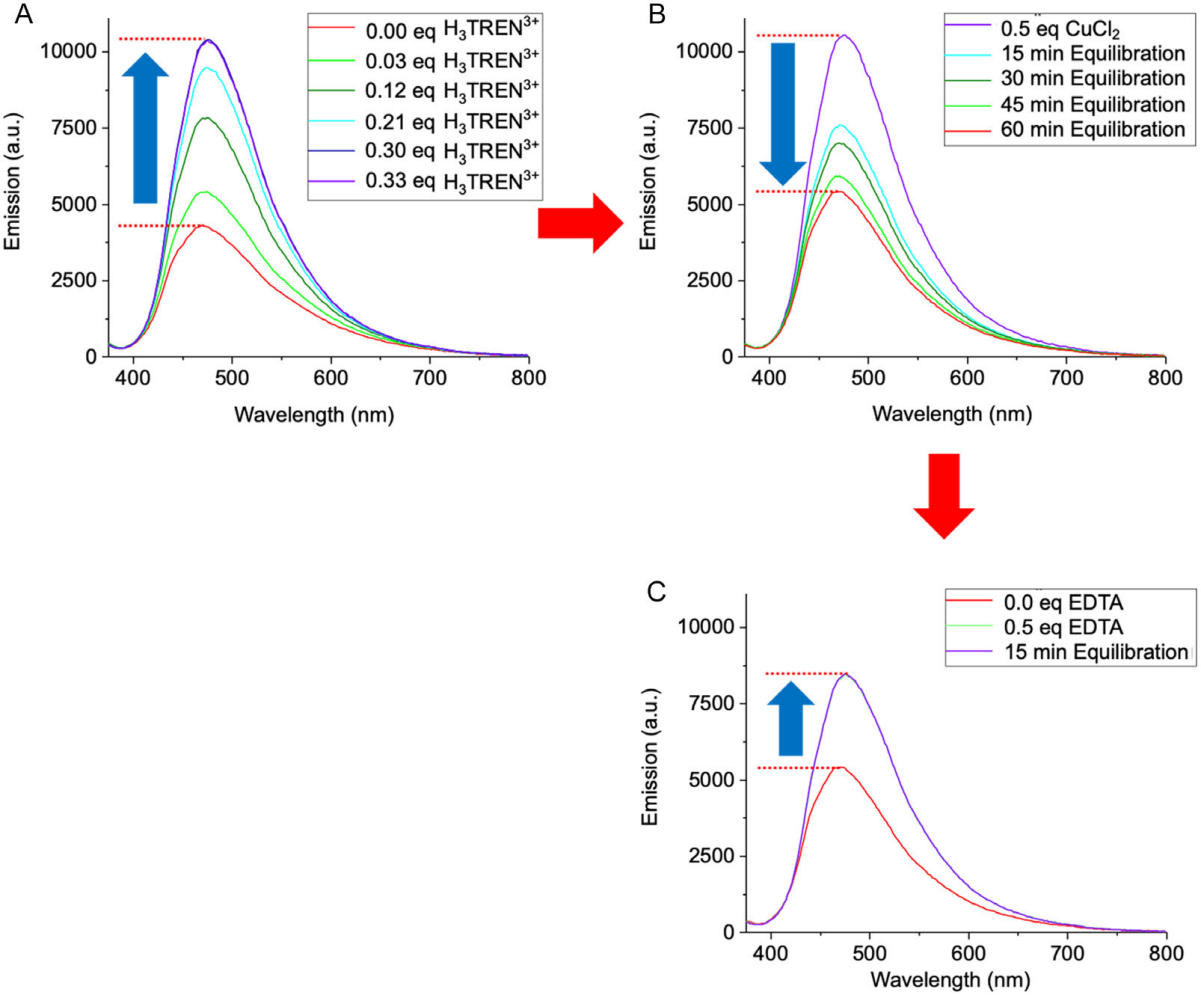
Signal transduction experiments in phase‐separated LUVs composed of the lipids DOPC/DPPC/Chol (1:2:1, *c* = 2 mM). The liposomes were doped with 5 mol% (0.1 mM) of receptor **1** and suspended in ultrapure water (pH 7.4, adjusted with NaOH). The sample solution (60 μL) was equilibrated at 25 °C (measurement temperature) for 45 min. A) H_3_TREN^3+^ (each *V *= 2.15 μL, *c *= 93 μM) was titrated to reach maximum signal intensity in the emission spectrum. B) Addition of CuCl_2_ (*V *= 2.32 μL, *c *= 1.3 mM) followed by equilibration for a total of 60 min resulted in a decrease in signal intensity. C) Titration with EDTA (*V *= 1.69 μL, *c *= 1.8 mM) led to Cu^2+^ complexation, reversing the Cu^2+^‐induced fluorescence decrease and enabling H_3_TREN^3+^ to reestablish receptor crosslinking and AIEE.

A general disadvantage of the receptor dimerization principle is basal fluorescence observed prior to messenger addition. It is commonly attributed to restricted rotation of the aromatic rotors in AIE luminophores. This effect likely arises from weak intermolecular interactions between AIE luminophores within locally enriched domains. Further evidence for the influence of weak intermolecular interactions on basal fluorescence comes from observations with non‐crosslinking messengers such as Cu^2+^ (Supporting Information, Figure S7D) and guanidinium, which disrupted receptor assemblies and increased their lateral separation, thereby lowering basal fluorescence—an effect consistent with literature reports on receptors in other chemical systems.^[^ ^37^, ^38^, ^39^ ^]^ Additionally, interactions within the hydrophobic core of the lipid matrix may further constrain rotor rotation. However, upon external messenger addition, a more compact arrangement of neighboring luminophores is induced almost immediately, which greatly enhances fluorescence emission. Background emission is always marked as a red starting line in the titration experiments (Figure 4–**7**); the ratio between fluorescence background and maximum induced emission is compiled in Figure S17, Supporting Information.

Why do receptors **1** and **2** show such a different signaling performance? This difference must be attributed to the presence of the estrone moiety in receptor **2**, which introduces additional steric and van der Waals interactions that enhance receptor clustering and facilitate a more stable AIEE response. Thus, while both receptors aggregate similarly, receptor **2**'s estrone moiety further optimizes molecular packing, leading to enhanced fluorescence response. This synergistic effect promotes a more efficient RIM process and, consequently, a stronger AIEE signal. In contrast, receptor **1**, while capable of messenger binding, likely forms aggregates with looser packing, allowing intramolecular rotations (e.g., around the C–S bond), which diminishes fluorescence enhancement.

Additionally, the inherent properties of the DOPC membrane, being less tightly packed than heterogeneous raft‐like membranes, may further reduce the stability of AIEE complexes for molecules like **1**. The absence of cholesterol and DPPC components, which can stabilize aggregates in raft‐like environments, likely exacerbates this effect.

### 2.3 Investigation of Signal Transmission in Raft‐Like Membranes

Building upon the findings from homogeneous DOPC membranes, further experiments were conducted in raft‐like lipid environments composed of DOPC/DPPC/cholesterol (1:2:1). These heterogeneous membranes contain both liquid‐ordered (Lo) and liquid‐disordered (Ld) phases, potentially offering an environment more conducive to receptor clustering and signal transmission. These experiments aimed at evaluating how phase separation and the resulting heterogeneous membrane organization influence receptor clustering and AIEE efficiency, compared to the fluid, homogeneous (Ld‐phase) environment provided by pure DOPC membranes.

To explore these effects, large unilamellar vesicles (LUVs) were prepared from DOPC/DPPC/cholesterol incorporating 5 mol% of receptor **1** or **2**. This system provided a controlled heterogeneous lipid environment, enabling a direct comparison with the results from homogeneous membranes. Importantly, apart from the membrane composition, all other experimental parameters, including messenger concentrations, and aqueous environment, were kept identical to ensure that any observed differences in signal transmission were solely due to phase separation effects. The goal was to determine whether receptor aggregation and signal amplification are influenced by phase separation model membranes. Experiments were initially conducted in ultrapure water, later also in HEPES buffer.

Upon stepwise addition of several 0.03 equivalent aliquots of H_3_TREN^3+^, receptor **1** exhibited a substantial fluorescence increase (**Figure** **5**). After the addition of 0.15 equivalents, fluorescence intensity had already increased by a factor of 2.3, reaching a maximum 2.6‐fold enhancement at 0.27 equivalents. This enhancement was much stronger than in homogeneous DOPC membranes, where receptor **1** exhibited minimal fluorescence changes upon messenger addition. It suggests that phase separation not only support receptor clustering but is a crucial factor in enabling AIEE. Similarly, titrations with H_2_DET^2+^ and H_2_EDA^2+^ led to an initial rapid fluorescence increase upon the addition of 0.25 equivalents, reaching a maximum 3.2‐fold (Figure 5B) and 1.8‐fold (Figure S14C, Supporting Information) fluorescence enhancement.

For estrone receptor **2** (Figure S14D–F, Supporting Information), fluorescence responses followed similar trends, but with slightly lower signal amplification compared to receptor **1**. At the end of the titration, a 1.8‐fold increase in fluorescence intensity was recorded for H_3_TREN^3+^, while H_2_DET^2+^ induced a 2‐fold enhancement and H_2_EDA^2+^ a 1.8‐fold increase.

The above results for receptor **1** highlight the critical role of phase separation in promoting receptor clustering and enhancing signal amplification. Based on spectroscopic evidence from related unpublished systems, we assume that receptors preferentially localize within the Ld‐phase of phase‐separated membranes, where steric constraints hinder their integration into the highly ordered Lo‐phase (raft‐like domains). Unlike the Ld‐phase in homogeneous DOPC membranes, the Ld‐phase in raft‐like membranes is spatially constrained by surrounding Lo‐domains. This spatial restriction likely increases the effective receptor concentration, facilitating receptor aggregation and optimizing signal transmission.

In contrast, receptor **2**, containing the estrone moiety, displayed the highest fluorescence response in homogeneous DOPC membranes but a somewhat lower response in raft‐like membranes. This counterintuitive trend suggests that while the estrone moiety enhances AIEE efficiency in less densely packed environment, the structural constraints of raft‐like membranes may limit its induced aggregation potential. These findings illustrate the complex interplay between receptor structure and membrane organization in modulating AIEE efficiency and signal transduction dynamics.

In order to imitate natural conditions, the signal transduction experiments were finally repeated in HEPES buffer (**Figure** **6**). In this competitive medium, much higher messenger concentrations were needed to reach the same AIEE effect. Maximum (≈twofold) signal enhancement was reached with 5 eqs. H_3_TREN^3+^ and 25 eqs. H_2_DET^2+^. In this experiments H_3_TREN^3+^ is superior to H_2_DET^2+^, most likely due to its multivalent character leading to more powerful oligomerization of the bisphosphonate head groups by electrostatic attraction. Additional titrations were conducted in MES buffer and in salt solution, as detailed in the Supporting Information (Figure S10 and S12). Further spectra for receptor **2** and titrations with H_2_EDA^2+^ are also provided (Figure S11D–F, Supporting Information). For clarity, only the most representative titration of receptor **1** in HEPES buffer is presented here.

### 2.4 Dynamic Modulation of the AIEE by Cu^2+^ and EDTA

The reversibility of the AIEE‐based signal transduction system was finally demonstrated using a sequence of ligand additions (**Figure** **7**). Initial receptor crosslinking and fluorescence enhancement were induced by titration with H_3_TREN^3+^, confirming receptor aggregation as the primary mechanism of signal amplification. Subsequent addition of Cu^2+^ led to a gradual decrease in fluorescence intensity, indicating receptor dispersion due to competitive bisphosphonate‐Cu^2+^ interactions. Most likely, a 1:1 complex is formed because saturation occurs at exactly 1 equivalent of added copper salt. Finally, titration with EDTA effectively reversed this effect by chelating Cu^2+^, thereby restoring the receptor's ability to reaggregate via H_3_TREN^3+^‐mediated crosslinking and recovering the AIEE signal. This reversibility highlights the dynamic nature of the system and its potential for controlled signal modulation.

## 3 Summary

In summary, we have introduced a new simplified signal transduction system which allows direct monitoring of receptor clustering by means of an aggregation‐induced emission. It employs the RTK principle and requires only one transducer molecule. A TM unit is equipped with a bisphosphonate head group for recognition of the incoming primary messenger signal. On the other end, a hydrophobic luminophore is attached, which is brought into close proximity of other AIE units by the bridging polyammonium cation. Receptor clustering by tight intermolecular interaction inside the membrane restricts bond rotations and leads to a powerful fluorescence emission.

In our systematic study several unique features of this AIE transmembrane approach became apparent: Very short transduction times indicate instantaneous signaling. This observation proves that recognition and lateral motion are fast processes, which do not slow down signal transduction. Closely related systems with a coupled chemical reaction are 50–100‐fold slower and reach their maximum signal after 10–15 min reflecting the reaction kinetics at the membrane surface. Turn‐on fluorescence from AIE‐luminophores indicate efficient receptor clustering, depending on lipid composition, TM receptor structure and concentration as well as ionic strength. At optimum receptor loading (5 mol%), substoichiometric messenger concentrations produce the maximum effect due to efficient multivalent receptor recognition; signaling efficiency directly depends on the number of cationic arms in the primary oligoamine messenger, indicating electrostatic attraction between messenger and receptor head group.

## 4 Conclusion

These findings hold promise for the construction of more advanced sensors and signaling systems employing the RTK principle with double steroid transmembrane units and bisphosphonate recognition head groups. Applications for induced conjugate addition reactions in the interior of syncells are underway in our laboratory.

## Conflict of Interest

The authors declare no conflict of interest.

## Supporting information

Supplementary Material

## Data Availability

The data that support the findings of this study are available in the Supporting Information of this article.
