# Supplementary material for: Monitoring Receptor Clustering by Aggregation‐Induced Emission
Source: Chempluschem. 2025 Sep 3;90(11):e202500286. doi: 10.1002/cplu.202500286 (PMC12605628; doi:10.1002/cplu.202500286)
Supplement: Supplementary file 1 — Supplementary Material [file CPLU-90-e202500286-s001.pdf]

Supporting information

Monitoring Receptor Clustering by Aggregation-Induced Emission

Robert Bekus*, Kevin Rudolph, Steffen Riebe, Jens Voskuhl, Thomas Schrader

1. General Information

Unless stated otherwise, all chemicals and solvents were used as received from commercial suppliers (Acros Organics, Alfa Aesar, ABCR, Carl Roth, Fisher Scientific, Merck, Sigma-Aldrich, TCI) without further purification. Tetrahydrofuran (THF) was freshly dried over sodium/benzophenone ketyl and distilled before use. Lipids were primarily obtained from Avanti Polar Lipids. Ultrapure water for the liposome experiments was obtained from a water purification system (ELGA, Purelab Classic UV). Liposomes were prepared as detailed below using an Avestin "LiposoFast" extruder with polycarbonate membranes (200 nm pore size, Whatman® Nuclepore).

Column chromatography for the purification of synthesized compounds was performed using silica gel 60 (40–63 µm, 230–400 mesh) from Merck or Macherey-Nagel. Thin-layer chromatography (TLC) was performed using Polygram® TLC plates (0.2 mm, SIL G/UV254) from Macherey-Nagel. When required, ALUGRAM® SIL G UV254 aluminum plates (0.2 mm, Macherey-Nagel) were used instead. In some cases, a potassium permanganate solution with the following composition was used as a staining reagent for TLC plates: 1.5 g $KMnO_4$, 10 g K_2CO_3, 1.25 mL of a 10% NaOH solution and 200 mL of water. In all other cases, detection was performed under UV light at a wavelength of 254 nm.

Nuclear magnetic resonance (NMR) spectra were recorded on a Bruker Avance HD 600 [^{1}H: 600 MHz, ^{13}C: 151 MHz and ^{31}P: 243 MHz] or a Bruker DMX 300 spectrometer [^{1}H: 300 MHz, ^{13}C: 75 MHz] spectrometer. Measurements were taken at room temperature, using deuterated chloroform ($CDCl_3$), deuterated methanol (CD_3OD) or deuterated dimethyl sulfoxide (DMSO-d_6) as solvents. For NMR spectra of the final products, a 2:1 mixture of $CDCl_3$ and CD_3OD was used. Chemical shifts (δ) are reported in ppm, referenced to the residual protium (^{1}H) signal in ^{1}H NMR or the ^{13}C resonance of the respective deuterated solvents in ^{13}C NMR. Coupling constants (*J*) are reported in Hertz (Hz). High -resolution mass spectra were obtained using a Bruker maXis 4G UHR-TOF or a Q Exactive Plus mass
spectrometer (Thermo Scientific, Bremen) equipped with an Orbitrap analyzer. In particular, dianionic end products were analyzed on the latter.

2. Synthetic procedures

2.1 Synthesis and Preparation of Polyammonium Messengers

To investigate artificial signal transduction, three compact polyamines – EDA (ethylenediamine), DET (diethylenetriamine), and TREN (tris(2-aminoethyl)amine) – were utilized as messengers. These polyamines were converted into their hydrochloride salts by reacting their ethanolic solutions with concentrated hydrochloric acid (37% HCl).[1] The resulting ammonium salts were precipitated, isolated as solids, and dried. This step was performed primarily for purification and stabilization of the polyamines, as amino groups are prone to oxidation. Aqueous solutions of these polyammonium salts were prepared using ultrapure water for the experiments.

Due to the pKa values of secondary (pKa 4.42)[2] and tertiary (pKa 2.60 in KNO_3 solution, 1.57 in NaCl solution)[3] amines, DET likely carries two positive charges, while TREN carries three positive charges at pH 7.4.[4] These partial protonation states result from unfavorable intramolecular repulsions between protonated NH^+ groups, caused by the short C_2 spacers. [5] The chemical structures and net charges of EDA, DET, and TREN are depicted in Figure 1.

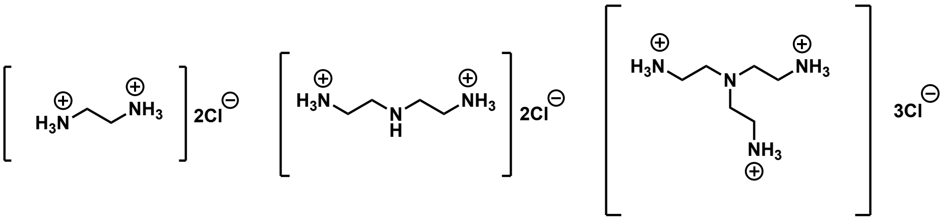

H_2EDA^{2+} pK_s 9.93 (+1), 7.11 (+2)

H_2DET^{2+} pK_s 10.02 (+1), 9.21 (+2), 4.42 (+3)

H_3TREN^{3+} pK_s 11.00 (+1), 10.26 (+2), 9.17 (+3)

Fig. 1: Lewis structures and net charges of the polyamines EDA, DET and TREN at pH 7.4.

2.2 Synthesis and Preparation of AIE receptors

The compounds **A**-**C** (Fig. 2) have been synthesized according to known literature procedures. **A**[6], **B**[7] and **C**[7]. The estrone based luminophore **4** was described by us beforehand and synthesized accordingly.[8] The procedure for compound **7** is provided below.

The synthetic route of the receptors is depicted in Fig. 3. Only compounds **D**[9] and **E**[10] are known from the literature, while the procedures for all other compounds are described herein.

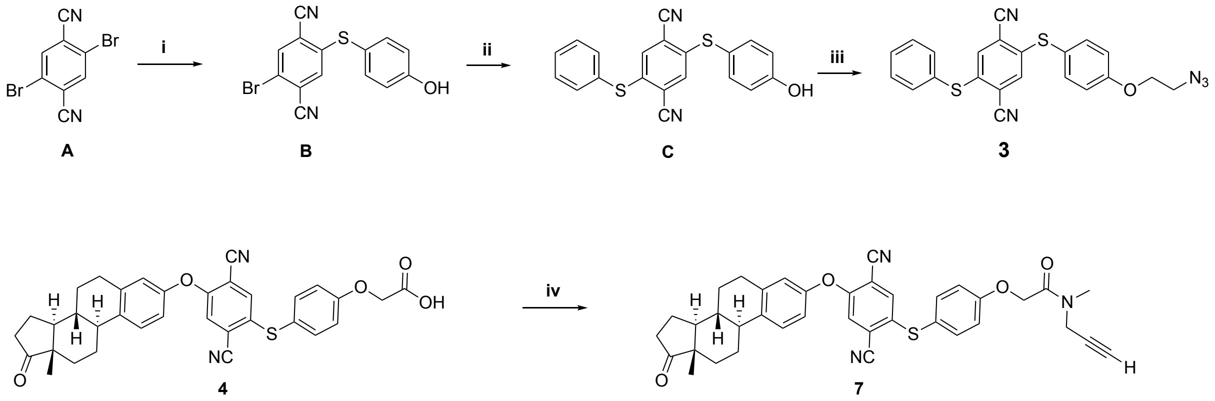

Fig. 2: Synthetic route to the azide **3** and estrone based luminophore **7**: **i**) K_2CO_3, 4-mercaptophenol, 50°C, 2h. **ii**) K_2CO_3, thiophenol, 45°C, 6h. **iii**) K_2CO_3, azidoethyl p-toluenesulfonate, 45°C, 4h. **iv**) EDC·HCl, 4-DMAP,rt, 24h.

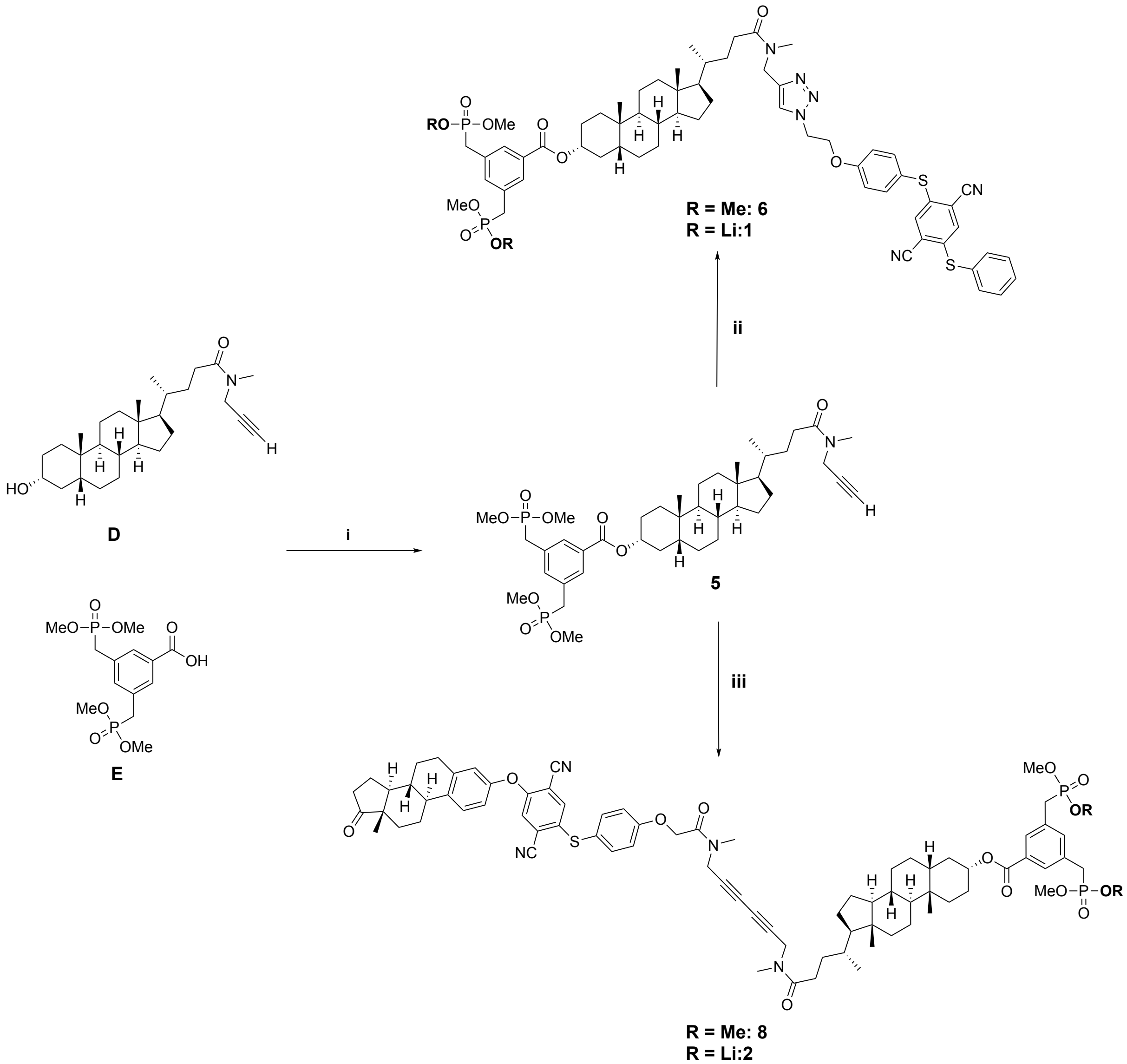

Fig. 3: Synthetic route to AIE receptors **1** and **2**: **i**) Esterification-based conjugation of bisphosphonate **E** to *N*-methylpropargylamide **D**: HCTU, Cl-HOBt, DCM/DMF, rt, 24 h, 40%. **ii**) Click Reaction: $Cu(CH_3CN)_4PF_6$, TBTA, DCM, rt, overnight, 75%; LiI-mediated demethylation: LiI, THF, reflux, overnight, quantitative. **iii**) Glaser-Hay Coupling: CuCl, DCM, TMEDA, O_2, rt, 5 h, 52%; LiI-mediated demethylation: LiI, THF, reflux, overnight, quantitative.

2-((4-(2-azidoethoxy)phenyl)thio)-5-(phenylthio)terephthalonitrile) (3)

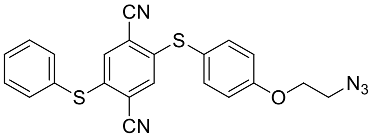

Compound **3** (100 mg, 0.28 mmol) was dissolved in 10 mL of dry DMF and K_2CO_3 (116 mg, 0.84 mmol) were added in one portion. The obtained solution was stirred for 30 minutes and azido-ethyltosylate[11] (79 mg, 0.31 mmol) were added and heated to 45 °C for 4 h. The reaction was quenched with water and the obtained solid was filtered yielding the desired compound **3** as a yellow solid. (Yield 113 mg, 93%).

^{1}H NMR (300 MHz, DMSO-d$_6$) δ = 7.75 (*s*, 2H), 7.57 (*d*, *J* = 8.7 Hz, 3H), 7.46 (*s*, 8H), 7.37 (*s*, 2H), 7.13 (*d*, *J* = 8.7 Hz, 4H), 4.29 – 4.21 (*m*, 4H), 3.73 – 3.64 (*m*, 4H).

^{13}C NMR (75 MHz, DMSO-d$_6$) δ = 159.65, 142.86, 137.55, 136.49, 135.87, 132.72, 132.16, 131.04, 130.15, 129.12, 127.99, 125.48, 119.22, 117.80, 116.57, 114.83, 67.08, 49.48.

IR (ATR): 2932, 2876, 2224, 2102, 1593, 1572, 1493, 1472, 1449, 1439, 1410, 1344, 1302, 1287, 1248, 1217, 1177, 1128, 1101, 1053, 1042, 1013, 907, 891, 882, 853, 833, 800, 752, 721, 689 675, 644, 615.
HR-ESI-pos (ACN): m/z = 452.0609. (calcd. 452.0610 for $[C_{22}H_{15}N_5OS_2Na]^+$).

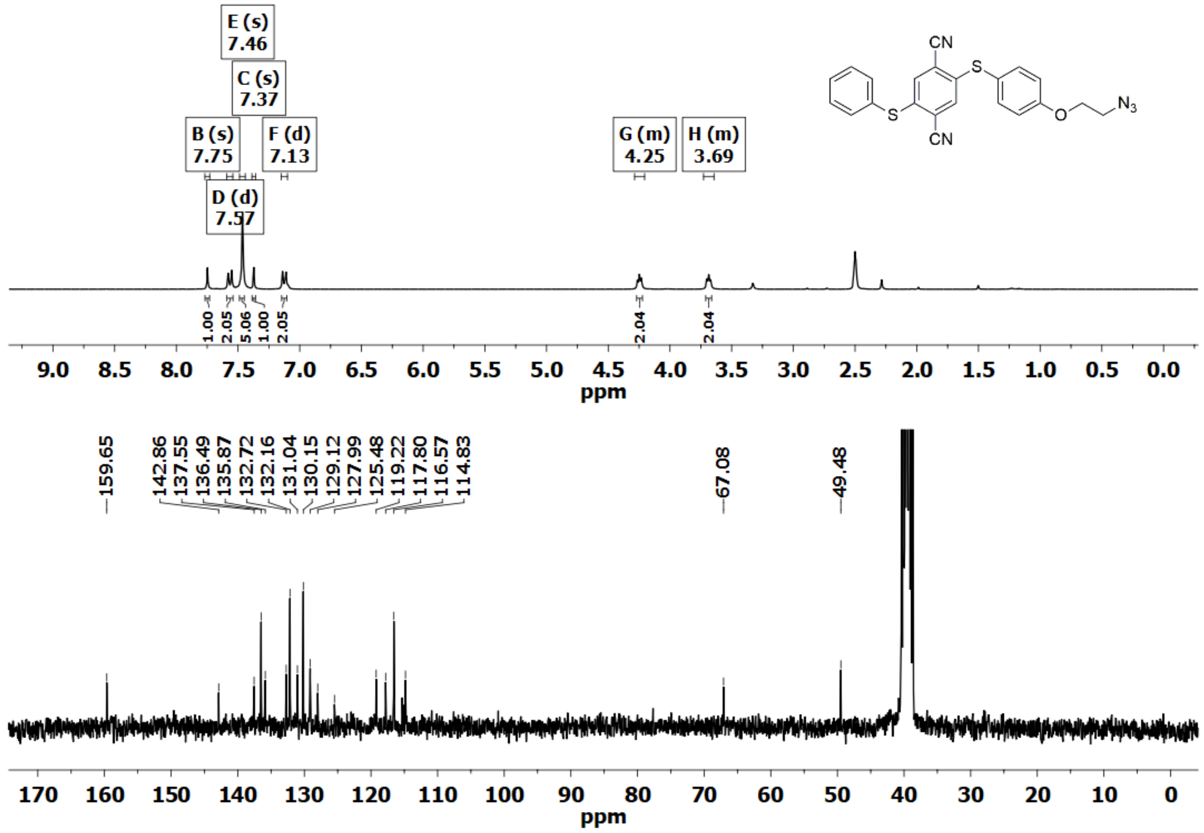

Fig. S1: ^{1}H NMR (300 MHz, DMSO-d6) and ^{13}C NMR (75 MHz, DMSO-d6).

Compound 7

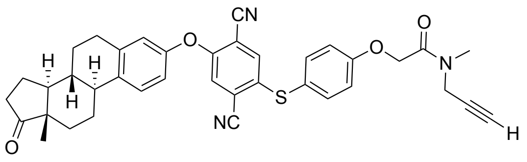

In a 10 mL round-bottom flask, estrone-luminophore **4** (50.0 mg, 86 µmol), *N*-methylpropargylamine (21.9 µL, 259 µmol), 4-DMAP (5.25 mg, 43 µmol) and EDC·HCl (36.3 mg, 189 µmol) were dissolved in dry dichloromethane (1.50 mL). The colorless reaction solution was stirred at room temperature for 24 h. The reaction mixture was then diluted with dichloromethane (10 mL) and washed with 1M HCl solution followed by deionized water (1x). The organic phase was dried over $MgSO_4$ and the solvent was removed under reduced pressure using a rotary evaporator. The residue (63 mg) was purified twice by column chromatography (1st: SiO_2, DCM/MeOH 160:1; 2nd: SiO_2, cyclohexane/ethyl acetate 1:1). Compound **7** (37 mg, 58.8 µmol, 68%) was obtained as an isomeric mixture in the form of a yellow solid.

^{1}H NMR (600 MHz, $CDCl_3$) δ (ppm) = 7.46 (d, *J* = 8.8 Hz, 2H), 7.34 (d, *J* = 8.5 Hz, 1H), 7.24 (s, 1H), 6.99 – 7.06 (m, 3H), 6.83 (dd, *J* = 8.5 Hz, *J* = 2.7 Hz, 1H), 6.79 (d, *J* = 2.7 Hz, 1H), 4.81 (s, 0.7H), 4.76 (s, 1.2H), 4.25 (d, *J* = 2.5 Hz, 1.2H), 4.19 (d, *J* = 2.5 Hz, 0.8H), 3.17 (s, 1.8H), 3.05 (s, 1.1H), 2.89-2.93 (m, 2H), 2.51 (dd, *J* = 19.0 Hz, *J* = 8.8 Hz, 1H), 2.38-2.45 (m, 1H), 2.36 (t, *J* = 2.5 Hz, 0.3H), 2.27 – 2.37

(m, 1H), 2.25 (t, *J* = 2.5 Hz, 5H), 2.20-2.11 (m, 1H), 2.01-2.10 (m, 2H), 1.98 (dt, *J* = 12.8 Hz, 3.5 Hz, 1H), 1.44-1.68 (m, 6H), 0.94 (s, 3H,).

^{13}C NMR (151 MHz, $CDCl_3$) δ (ppm) = 220.59, 167.18, 166.95, 159.46, 158.12, 151.61, 139.48, 138.11, 137.03, 136.45, 133.94, 127.54, 121.77, 120.38, 117.55, 116.62, 116.59, 115.32, 114.23, 107.87, 77.91, 77.50, 73.42, 72.46, 67.43, 67.20, 50.39, 47.92, 44.11, 38.81, 37.94, 36.66, 35.82, 33.69, 31.52, 29.44, 26.89, 26.21, 25.78, 21.58, 13.84.

IR (ATR): 3279, 3094, 3065, 3032, 2957, 2922, 2853, 2232, 2116, 1780, 1732, 1663, 1589, 1545, 1491, 1466, 1406, 1373, 1260, 1240, 1209, 1177, 1157, 1084, 1055, 1009, 930, 914, 883, 824, 800, 725, 706, 664, 646.

HRMS calc. for $[C_{38}H_{36}N_3O_4S]^+$: 630.2421; found: 630.2417.

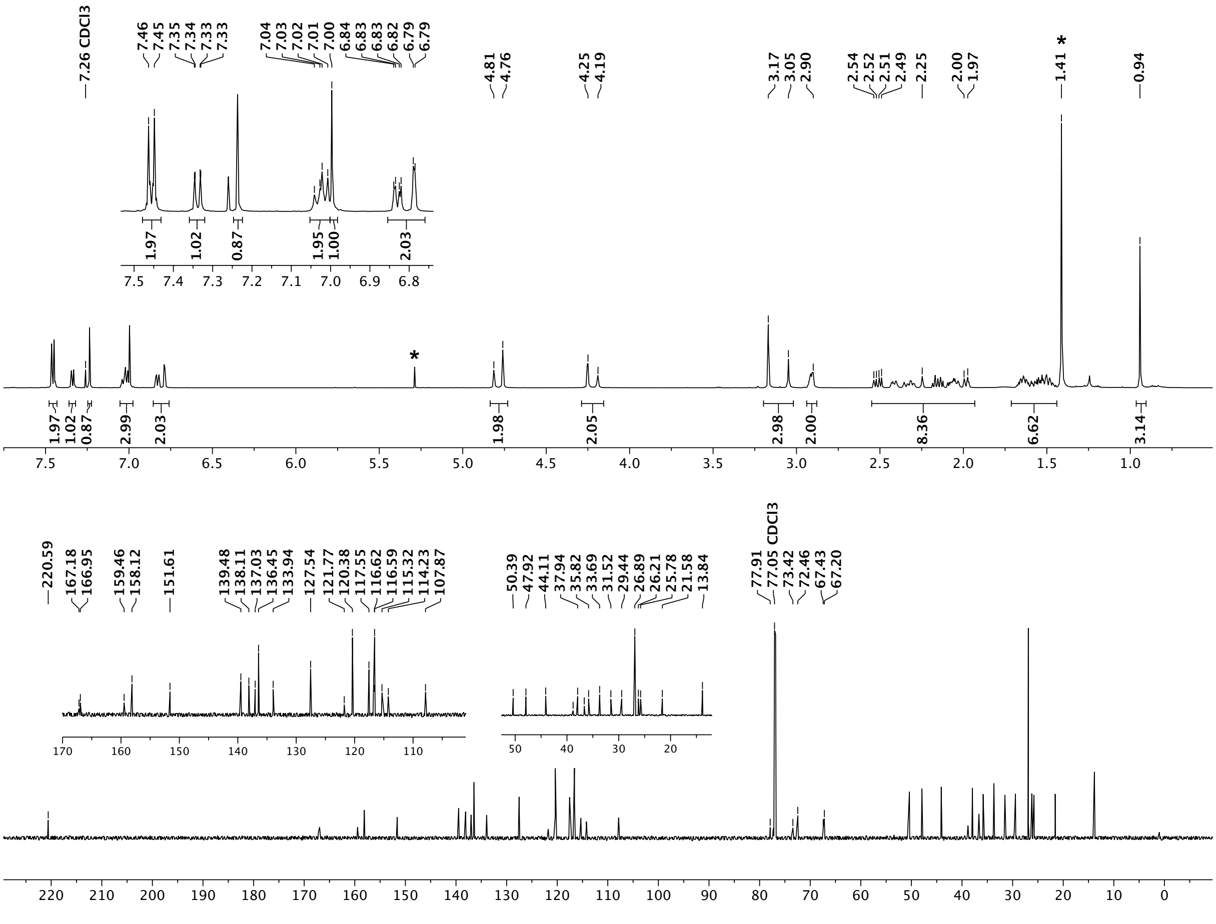

Fig. S2: ^{1}H NMR (600 MHz, $CDCl_3$) and ^{13}C NMR (151 MHz, $CDCl_3$). *: DCM and Cyclohexane.

Compound 6

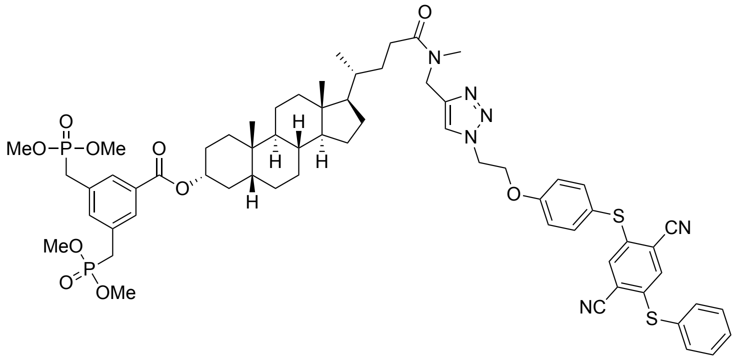

In a 10 mL single-neck flask flushed with argon, bisphosphonate **5** (18 mg, 23.3 µmol) and AIE fluorophore **3** (10 mg, 23.0 µmol) were dissolved in dichloromethane (6 mL). To this yellow solution, $Cu(CH_3CN)_4{\cdot}PF_6$ (1.48 mg, 3.96 µmol) and TBTA (2.10 mg, 3.96 µmol) were added. The reaction mixture was stirred at room temperature for 24 h. The reaction mixture was then diluted with dichloromethane (10 mL), washed with a saturated ammonium chloride solution and dried over $MgSO_4$. The solvent was removed under reduced pressure using a rotary evaporator, yielding a yellow-green crude product (51 mg). Purification was performed by column chromatography (SiO_2, DCM/MeOH gradient 40:1 → 19:1). The fraction with R_f (DCM/MeOH 19:1) = 0.30 yielded compound **6** (21 mg, 17.4 µmol, 75%) as a yellow-green solid.

^{1}H NMR (600 MHz, CDCl$_3$) δ (ppm) = 7.84 (d, ^4J = 2.2 Hz, 2H), 7.79 (s, 0.7H), 7.61 (s, 0.2H), 7.41–7.49 (m, 7H), 7.17 (s, 1H), 7.01 (s, 1H), 6.96 (d, J = 8.7 Hz, 2H), 4.90–4.98 (m, 1H), 4.81 (t, ^3J = 5.0 Hz, 0.4H, H-57), 4.76 (t, J = 4.9 Hz, 1.5H), 4.54–4.66 (m, 2H), 4.41 (t, J = 4.9 Hz, 2H), 3.69 (dd, $^3J_{PH}$ = 10.9 Hz, J_{PH} = 1.2 Hz, 12H), 3.19 (d, $^2J_{PH}$ = 21.8 Hz, 4H), 3.11 (s, 2.2H), 3.07 (s, 0.2H), 2.98 (s, 0.1H), 2.96 (s, 0.4H), 2.14–2.38 (m, 2H), 0.98–2.13 (m, 29H in lithocholic acid), 0.86–0.96 (m, 6H), 0.62–0.67 (m, 3H).

^{13}C NMR (151 MHz, CDCl$_3$) δ (ppm) = 173.77, 165.63, 159.81, 142.96, 140.51, 137.21, 135.29, 134.33, 133.29, 132.28, 132.20, 131.91, 131.83, 130.46, 130.19, 129.84, 129.71, 129.67, 120.33, 116.67, 116.30, 115.38, 115.20, 114.88, 75.44, 66.56, 56.54, 56.12, 53.56, 53.14, 53.12, 53.09, 53.08, 42.97, 42.89, 42.09, 40.56, 36.00, 35.93, 35.65, 35.20, 34.75, 33.17, 32.43, 32.25, 31.13, 30.42, 28.42, 27.16, 26.85, 26.43, 24.33, 23.46, 21.02, 18.66, 12.19.

IR (ATR): 2924, 2853, 2228, 2101, 1709, 1638, 1593, 1493, 1451, 1402, 1377, 1323, 1304, 1244, 1209, 1177, 1105, 1024, 984, 901, 802, 754, 723, 691, 615.

HRMS calc. for $[C_{63}H_{79}N_6O_{10}P_2S_2]^+$: 1205.4769; found: 1205.4784.

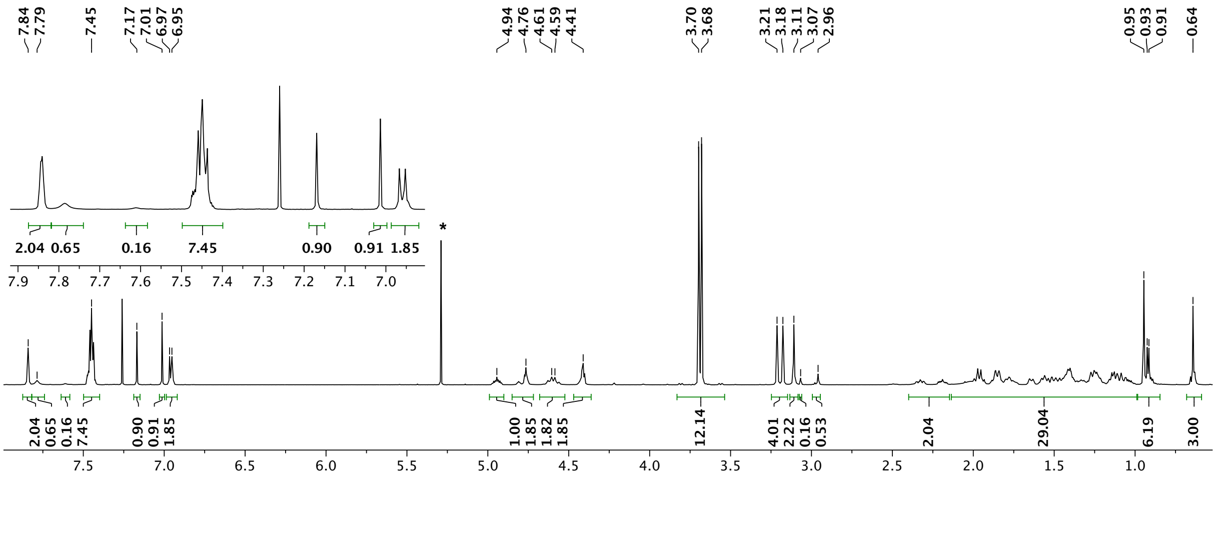

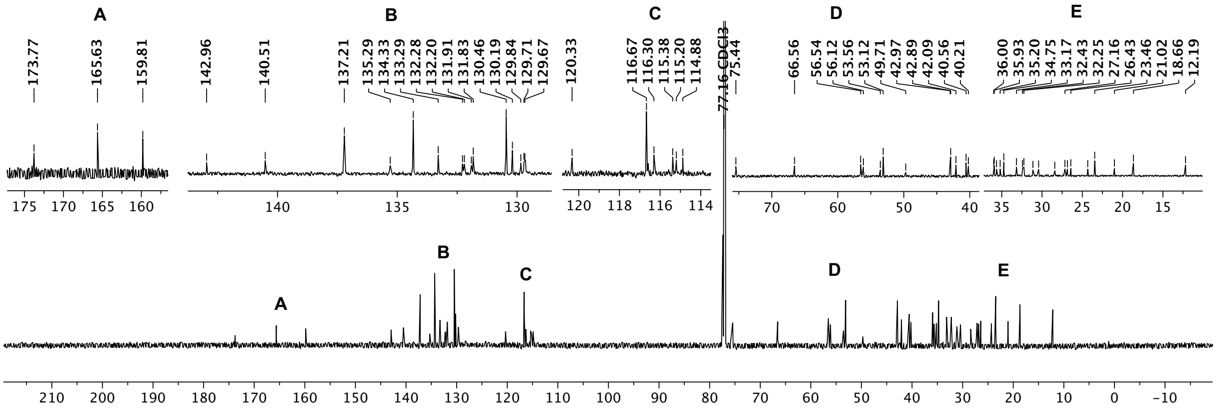

Fig.S3: ^{1}H NMR (600 MHz, $CDCl_3$) and ^{13}C NMR (151 MHz, $CDCl_3$). *: DCM.

Receptor 1

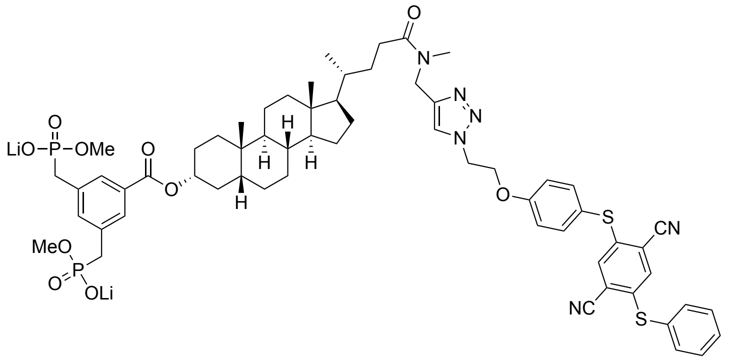

In a flame-dried 10 mL two-neck flask, AIE receptor **1** (10 mg, 8.30 µmol) was dissolved in dry tetrahydrofuran (5 mL) and lithium iodide (2.78 mg, 20.74 µmol) was added. The solution was heated to 80 °C and stirred at this temperature for 24 h. The reaction mixture was then cooled to room temperature and the solvent was removed under reduced pressure using a rotary evaporator. After drying under high vacuum, compound **1** (10 mg, 8.30 µmol, quantitative yield) was obtained as a yellow crystalline solid.

^{1}H NMR (600 MHz, $CDCl_3$/MeOD) δ (ppm) = 7.85 (s, 1H), 7.71 (br.s, 2H), 7.55 (br.s, 1H), 7.50 (s, 1H), 7.40–7.49 (m, 6H), 7.20 (s, 0.6H), 7.19 (s, 0.3H), 7.03 (s, 1H), 7.00 (d, ^3J = 8.8 Hz, 2H), 4.84–4.92 (m, 1H), 4.83 (t, J = 5.0 Hz, 0.4H), 4.79 (t, J = 5.0 Hz, 1.4H), 4.58–4.66 (m, 0.5H), 4.57 (s, 1.5H), 4.44 (t, J = 5.0 Hz, 0.5H), 4.42 (t, J = 5.1 Hz, 1.4H), 3.52 (d, $^3J_{PH}$ = 10.5 Hz, 6H), 3.08 (s, 2.3H), 2.97 (d, $^2J_{PH}$ = 20.5 Hz, 4H), 2.93 (s, 0.7H), 2.32–2.40 (m, 1H), 2.13–2.26 (m, 1H), 0.94 (s, 3H), 0.92 (d, J = 6.5 Hz, 2H), 0.89 (d, J = 6.5 Hz, 0.7H), 0.75–2.03 (m, 29H, in lithocholic acid), 0.66 (s, 0.3H), 0.63 (s, 1.9H), 0.62 (s, 0.7H).

^{13}C NMR (151 MHz, $CDCl_3$/MeOD) δ (ppm) = 174.56, 174.32, 166.68, 159.61, 143.70, 143.50, 142.79, 139.95, 136.73, 135.56, 135.37, 133.70, 133.16, 131.49, 129.99, 129.91, 129.68, 129.55, 127.81, 124.23, 123.04, 119.71, 116.28, 116.23, 116.04, 114.80, 114.61, 114.40, 74.86, 66.14, 56.12, 55.65, 51.05, 51.01, 49.39, 42.45, 42.39, 41.73, 40.18, 39.79, 35.55, 35.38, 35.25, 34.72, 34.31, 34.05, 33.30, 33.18, 31.99, 30.76, 30.08, 27.89, 26.72, 26.35, 26.02, 23.84, 22.84, 20.53, 17.99, 17.96, 11.55.

^{31}P NMR (243 MHz, $CDCl_3$/MeOD) δ (ppm) = 21.33. IR (ATR): 2926, 2857, 2228, 2114, 1707, 1624, 1593, 1493, 1451, 1408, 1377, 1325, 1304, 1238, 1204, 1177, 1047, 986, 899, 833, 791, 725, 702, 613.

HRMS calc. for $[C_{61}H_{72}N_6O_{10}P_2S_2]^{2-}$: 587.2118; found: 587.2124.

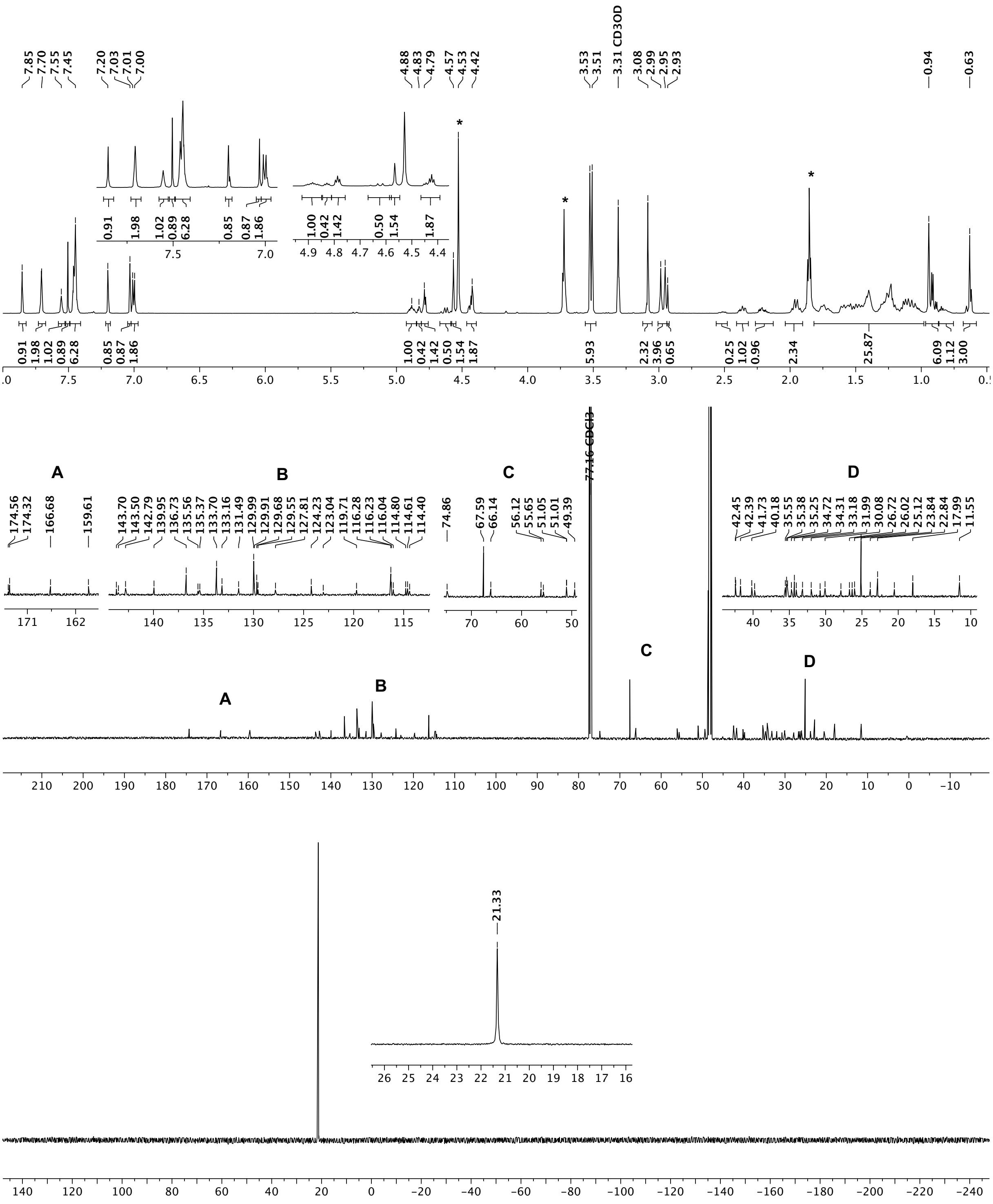

Fig. S4: ^{1}H NMR (600 MHz, $CDCl_3$/ MeOD), ^{13}C NMR (151 MHz, $CDCl_3$/ MeOD) and ^{31}P-NMR (243 MHz, $CDCl_3$/ MeOD). *: THF and H_2O.

Compound 8

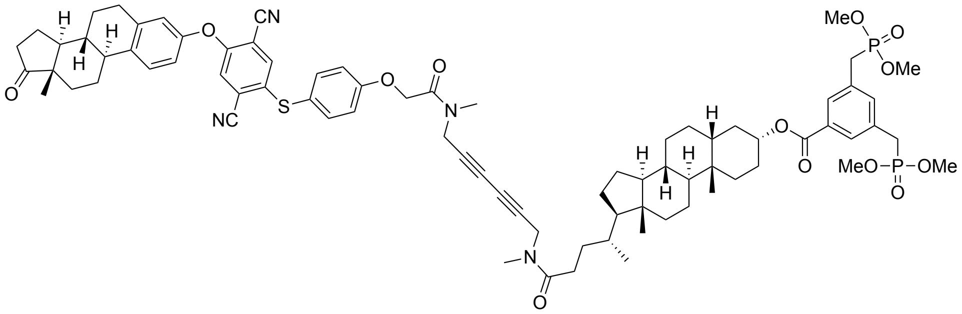

Under an argon atmosphere, bisphosphonate **5** (113.3 mg, 0.15 mmol) and estrone based luminophore **7** (46,0 mg, 0,07 mmol) were dissolved in dichloromethane in a 2:1 ratio, and copper(I) chloride (108,5 mg, 1.10 mmol, 5 equiv., relative to the total concentration of acetylene units) was added. The suspension was then treated with *N*,*N*,*N'*,*N'*-tetramethylethylenediamine (170 µL, 127,3 mg, 1.10 mmol, 5 equiv., relative to the total concentration of acetylene units). The mixture was stirred until the copper(I) chloride was completely dissolved, turning the solution light green. The atmosphere above the reaction solution was carefully evacuated under reduced pressure and subsequently replaced with oxygen from a balloon. This procedure was repeated three times. During this process, the solution color changed from light green to deep blue. The reaction was stirred at room temperature for a total of 5 h, then diluted with dichloromethane and washed with a saturated ammonium chloride solution in a separatory funnel. The organic phase were dried over $MgSO_4$ and the solvent was removed under reduced pressure using a rotary evaporator. The green crude product (188 mg) was purified by column chromatography (SiO_2, DCM/MeOH gradient 40:1 → 19:1 → 10:1), yielding three reaction products (homo- and heterodimers) in the following order:

The first fraction with R_f (DCM/MeOH 40:1) = 0.29 gave the homodimer **7a** (12 mg, 9.54 µmol, 26% relative to **7**) as a yellow solid.

The second fraction with R_f (DCM/MeOH 19:1) = 0.35 gave the heterodimer **8** (54 mg, 38.0 µmol, 52% relative to **7**) as a yellow solid.

The third fraction with R_f (DCM/MeOH 10:1) = 0.25 yielded **5a** (58 mg, 37.0 µmol, 25% relative to **5**) as a white crystalline solid.

As expected in the asymmetric Glaser-Hay reaction, side products were observed. However, no further spectral characterization is provided in this document, as they are not relevant to the scope of this study.

Homodimeric byproduct derived from compound **7**

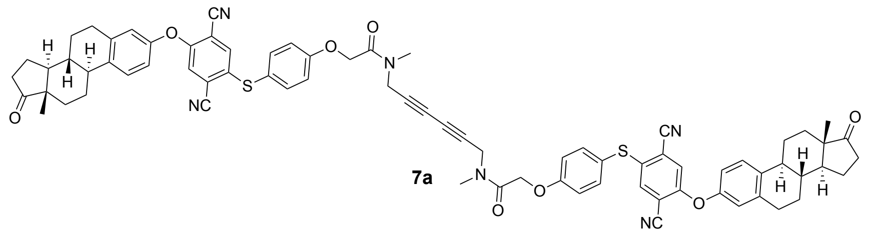

7a

Homodimeric byproduct derived from compound **5**

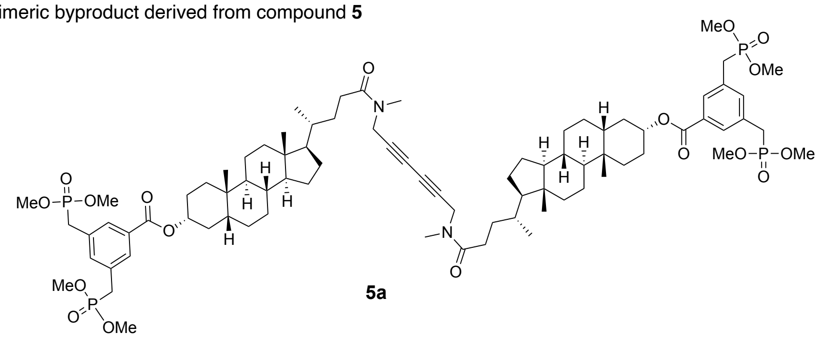

5a

Fig. 4: Homodimeric byproduct of **5** and **7** observed in the asymmetric Glaser-Hay reaction.

Spectral data of Compound **8**:

^{1}H NMR (600 MHz, CDCl$_3$) δ (ppm) = 7.80–7.85 (m, 2H), 7.41–7.47 (m, 3H), 7.32 (dd, ^3J = 8.7 Hz, ^4J = 1.1 Hz, 1H), 7.22 (s, 1H), 6.96–7.04 (m, 3H), 6.82 (dd, ^3J = 8.5 Hz, ^4J = 2.7 Hz, 1H), 6.77 (d, ^4J = 2.6 Hz, 1H), 4.90–4.96 (m, 1H), 4.77 (s, 0.6H), 4.74 (br.s, 1.3H), 4.23–4.33 (m, 3.4H), 4.12 (br.s, 0.6H), 3.67 (d, $^3J_{PH}$ = 10.8 Hz, 12H), 3.33 (br.s, 1H), 3.19 (d, $^2J_{PH}$ = 21.8 Hz, 4H), 3.11–3.15 (m, 2H), 3.00–3.07 (m, 3.1H), 2.96 (br.s, 0.7H), 2.86–2.92 (m, 2H), 0.98–2.54 (m, 42H in lithocholic acid and estrone), 0.89–0.95 (m, 9H), 0.64 (s, 3H).

^{13}C NMR (151 MHz, CDCl$_3$) δ (ppm) = 220.66, 173.47, 167.21, 167.05, 165.55, 159.47, 159.32, 158.21, 151.67, 139.56, 138.19, 137.05, 136.53, 135.27, 134.03, 132.22, 132.16, 132.09, 131.88, 131.85, 129.67, 129.62, 129.57, 127.61, 121.92, 120.45, 120.44, 117.63, 116.68, 116.66, 115.39, 114.29, 107.94, 75.38, 67.61, 67.17, 56.49, 56.09, 53.54, 53.13, 53.09, 53.05, 50.46, 47.99, 44.18, 42.84, 42.03, 40.50, 40.16, 39.63, 38.01, 37.42, 36.94, 35.89, 35.87, 35.57, 35.14, 34.81, 34.69, 34.03, 33.30, 32.36, 31.92, 31.59, 31.03, 30.28, 29.51, 28.33, 27.10, 26.79, 26.37, 26.28, 25.85, 24.28, 23.40, 21.66, 20.96, 18.60, 13.91, 12.14.

IR (ATR): 2928, 2864, 2349, 2324, 2234, 2100, 1736, 1711, 1649, 1591, 1547, 1512, 1493, 1466, 1402, 1375, 1325, 1306, 1240, 1207, 1179, 1159, 1109, 1053, 1026, 986, 903, 851, 802, 725, 692, 644.

HRMS calc. for [$C_{79}H_{96}N_4NaO_{13}P_2S$]$^+$: 1425.6062; found: 1425.6051.

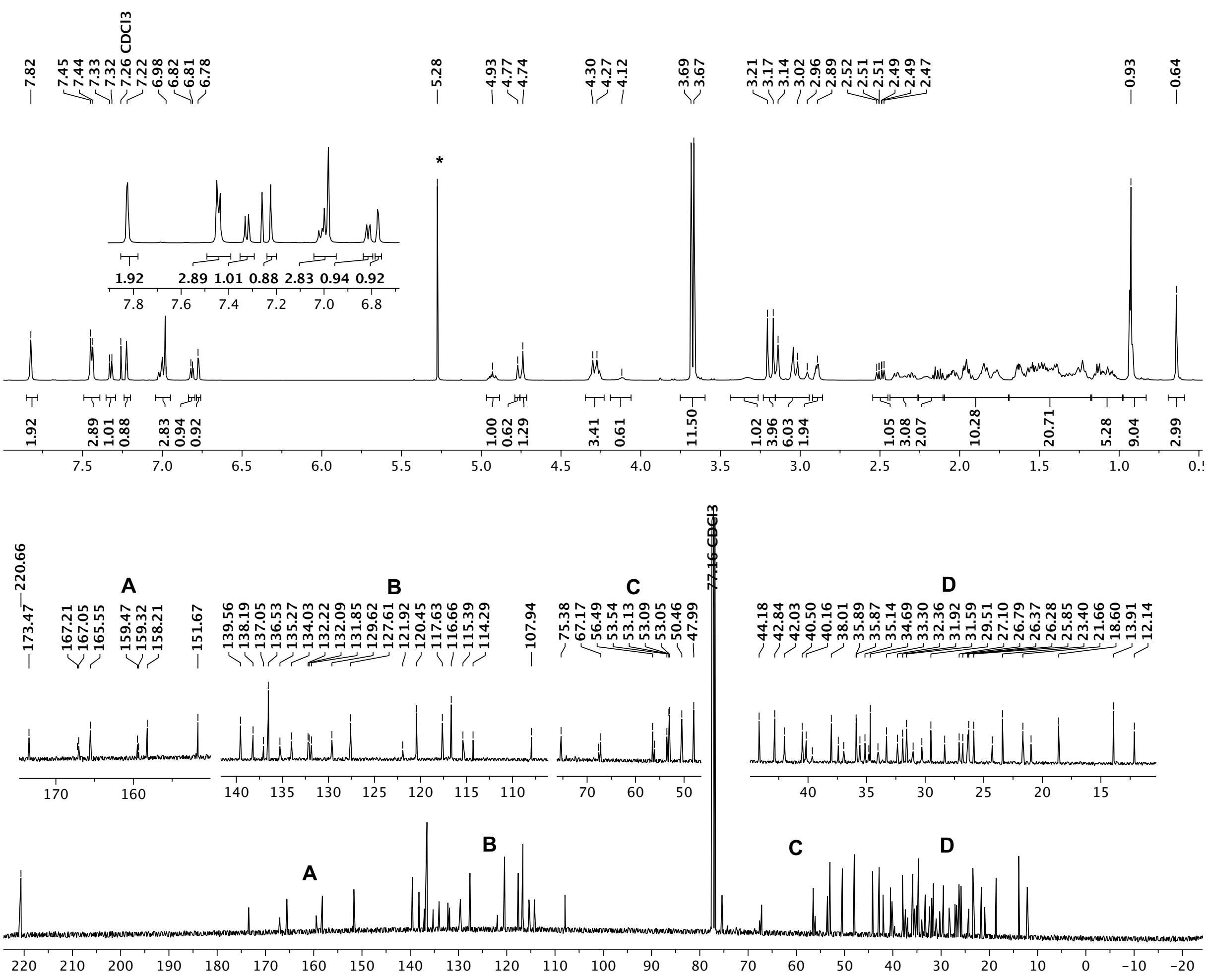

Fig. S5: ^{1}H NMR (600 MHz, $CDCl_3$) and ^{13}C NMR (151 MHz, $CDCl_3$). *: DCM.

Receptor 2

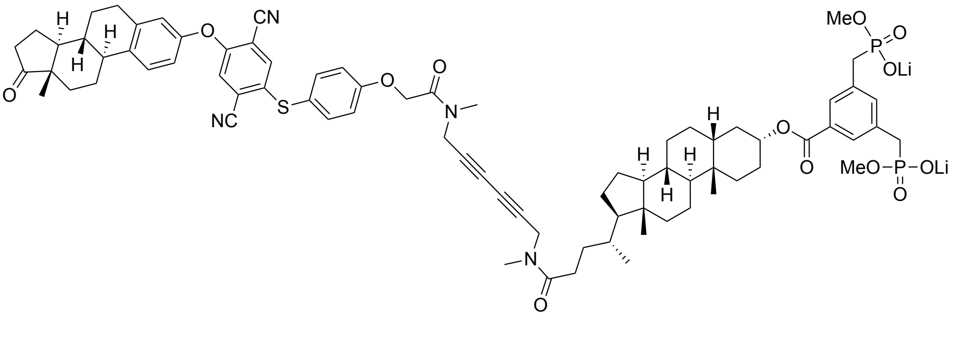

In a flame-dried 10 mL two-neck flask, AIE receptor 118 (20 mg, 14.2 µmol) was dissolved in dry tetrahydrofuran (10 mL), and lithium iodide (4.68 mg, 35.6 µmol) was added. The colorless solution was heated to 80 °C and stirred at this temperature for 24 h. The reaction mixture was then cooled to room temperature and the solvent was removed under reduced pressure using a rotary evaporator. After drying under high vacuum, compound **2** (20 mg, 14.2 µmol, quantitative yield) was obtained as an orange-yellow solid.

^{1}H NMR (600 MHz, $CDCl_3$/MeOD) δ (ppm) = 7.46 (s, 2H), 7.31 (s, 1H), 7.19–7.24 (m, 2H), 7.10 (d, ^2J = 8.6 Hz, 1H), 7.01–7.07 (m, 1H), 6.72–6.84 (m, 3H), 6.59 (dd, ^3J = 8.5 Hz, ^3J = 2.7 Hz, 1H), 6.56 (d, ^4J = 2.6 Hz, 1H), 4.6–4.67 (m, 1H), 4.58 (s, 0.7H), 4.55 (s, 1.3H), 3.91–4.11 (m, 4H), 3.27 (d, ^3J = 10.5 Hz, 6H), 2.91 (s, 0.6H), 2.89 (s, 1.2H), 2.87 (s, 0.7H), 2.83 (s, 1.3H), 2.78 (s, 0.3H), 2.77 (s, 0.7H), 2.72 (d, ^2J = 20.7 Hz, 5H), 2.64–2.67 (m, 2H), 0.68 (s, 4H), 0.69 (s, 5H), 0.48–2.30 (m, 46H in estrone and lithocholic acid), 0.40 (s, 3H).

^{13}C NMR (151 MHz, $CDCl_3$/MeOD): δ (ppm) = 222.21, 174.24, 174.07, 167.71, 167.56, 166.67, 159.13, 159.03, 157.92, 151.41, 139.25: 137.74, 136.71, 135.99, 135.55, 135.44, 135.38, 133.87, 129.88, 127.77, 127.22, 121.63, 120.14, 119.98, 117.15, 116.44, 116.27, 116.24, 114.84, 113.71, 107.55, 97.64, 74.82, 74.39, 73.35, 73.15, 72.35, 72.07, 71.39, 68.90, 68.79, 68.32, 67.90, 67.16, 66.68, 66.38, 66.10, 56.12, 55.78, 55.64, 51.02, 51.00, 50.98, 50.07, 43.78, 42.44, 41.70, 40.17, 39.93, 39.78, 39.02, 37.66, 36.96, 36.62, 35.53, 35.50, 35.26, 35.17, 34.69, 34.61, 34.54, 34.28, 34.02, 33.43, 33.35, 33.15, 32.86, 31.96, 31.11, 30.68, 29.94, 29.27, 29.04, 27.85, 26.70, 26.32, 26.00, 25.85, 25.40, 25.10, 23.83, 23.02, 22.81, 21.17, 20.51, 17.96, 13.34, 11.56, 11.53.

^{31}P NMR (243 MHz, $CDCl_3$/MeOD): δ (ppm) = 25.26.

IR (ATR): 2926, 2862, 2349, 2324, 2234, 2112, 2077, 1736, 1709, 1688, 1657, 1641, 1632, 1591, 1547, 1512, 1493, 1466, 1452, 1406, 1373, 1325, 1306, 1234, 1206, 1180, 1159, 1051, 986, 899, 831, 791, 725, 702, 644.

HRMS calc. for $[C_{77}H_{90}LiN_4O_{13}P_2S_1]^-$: 1379.5865; found: 1379.5863.

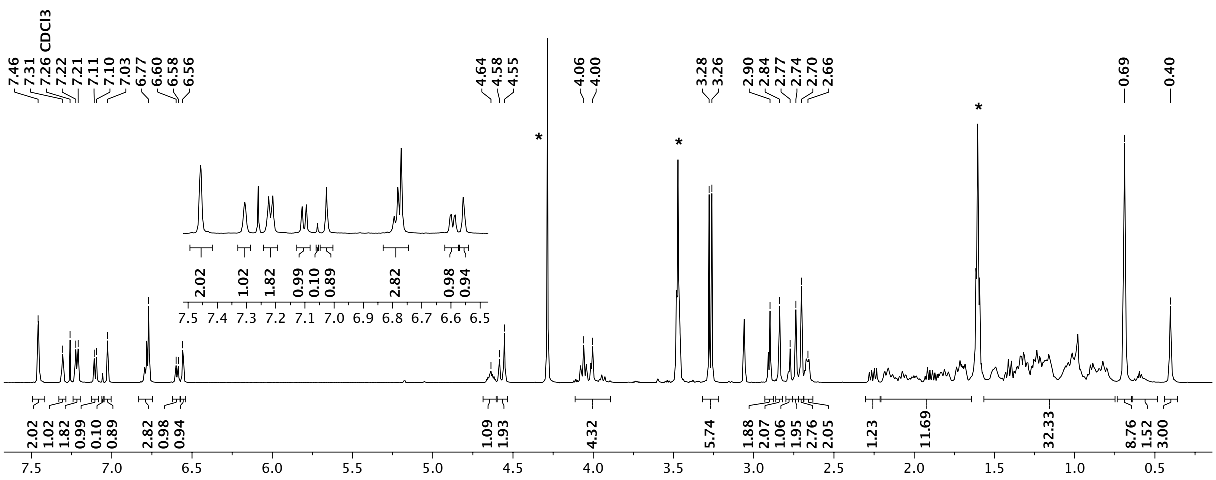

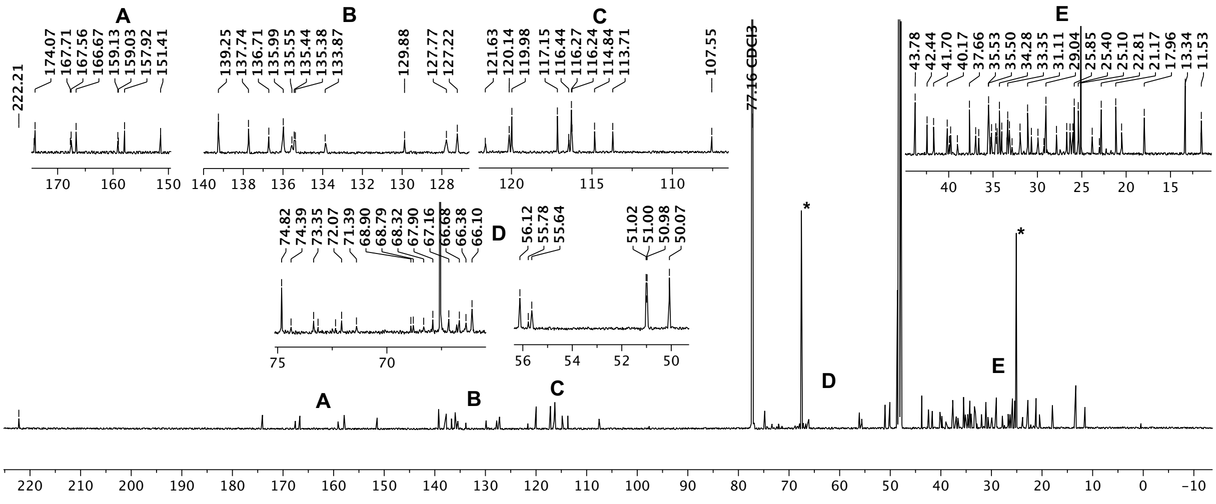

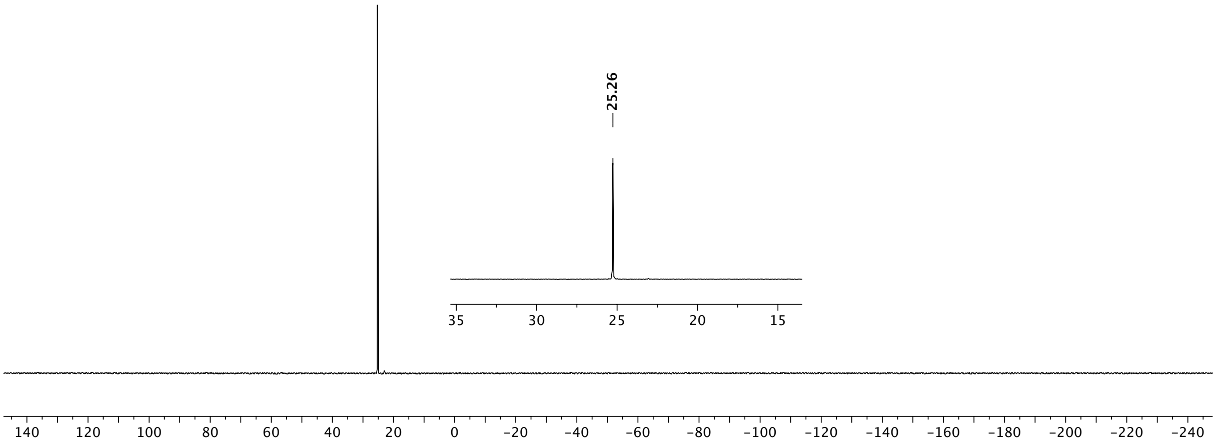

Fig. S6: ^{1}H NMR (600 MHz, $CDCl_3$/ MeOD), ^{13}C NMR (151 MHz, $CDCl_3$/ MeOD) and ^{31}P-NMR (243 MHz, $CDCl_3$/ MeOD). *: THF and H_2O.

3. General procedure for liposome preparation

Liposomes were prepared using the thin-film hydration method followed by extrusion to achieve large unilamellar vesicles (LUVs) [12], with minor modifications. The lipid compositions of the vesicles included:

1. Homogeneous membranes: Composed entirely of 1,2-dioleoyl-sn-glycero-3-phosphocholine (DOPC), representing the liquid-disordered (l_d) phase.

2. Raft-Like membranes: Composed of DOPC, 1,2-dipalmitoyl-sn-glycero-3-phosphocholine (DPPC), and cholesterol (Chol) in a 1:2:1 molar ratio to represent coexisting liquid-ordered (l_o) and l_d phases.[13]

To begin, stock solutions of the lipids (10 mg/mL) were prepared in chloroform, while the AIE receptors were dissolved in a chloroform/methanol (1:1) mixture. Receptor incorporation was achieved by mixing receptor and lipid solutions. The amphiphilic mixture was prepared in a 1.5 mL Eppendorf tube, containing, for example, DOPC, DPPC, and cholesterol in a 1:2:1 molar ratio, doped with 2.5, 5, or 10 mol% of a single type of AIE receptor. After thorough vortexing to ensure homogeneity, the solvent ($CHCl_3$/MeOH) was gently evaporated under a stream of argon. Residual solvent traces were removed by overnight drying under vacuum, yielding a thin lipid film.

The film was hydrated with 1.0 mL of ultrapure water or buffer solutions (e.g., MES, HEPES) to achieve a 2 mM lipid concentration. The resulting suspension was vortexed to ensure complete hydration, forming multilamellar vesicles (MLVs). To further homogenize the MLVs, the suspension underwent five freeze-thaw cycles.[14] Each cycle involved freezing the sample in liquid nitrogen for 2 minutes and then thawing it in a 65 °C water bath for 15 minutes, above the phase transition temperature (Tm). After the freeze-thaw process, the suspension was treated in an ultrasonic water bath at 65 °C for 5 minutes to uniformly disperse the lipids.

The MLVs were subsequently converted into large unilamellar vesicles (LUVs) with a uniform size distribution by extruding the lipid suspension through a polycarbonate membrane with a pore size of 200 nm. The extrusion process was repeated 21 times at 65 °C to ensure size homogenization. The resulting LUVs were transferred into a glass vial and stored at 4 °C until further use in receptor aggregation experiments.

4. UV/Vis and fluorescence spectroscopy

UV/Vis spectra were recorded at 20 °C using a Jasco V-550 spectrometer and quartz cuvettes with a path length of 1 cm. Fluorescence spectra were acquired using a TECAN SPARK spectrometer in 96-well microtiter plates. All measurements were performed at room temperature.

Fluorescence measurements were conducted to assess the emission intensity of AIE receptors before and after the addition of polyamine messengers. The experimental setup and titration conditions included:

1. Excitation wavelengths: 293 nm selectively induces emission from the AIE luminophores, whereas 230 nm leads to emission from both the AIE luminophores and the bisphosphonate groups.

2. Emission ranges: 270–650 nm, with specific monitoring at 472 nm (AIE luminophore emission) and 312 nm (bisphosphonate emission).

3. Messenger Titration: Incremental additions of H_2EDA^{2+}, H_2DET^{2+}, or H_3TREN^{3+}, each tested individually, were performed to evaluate their specific effects on fluorescence enhancement.

5. Calculation of ligand equivalents exemplified by a 5 mol% receptor doping

For the preparation of large unilamellar vesicles (LUVs) with a total lipid concentration of 2 mmol/L, 5 mol% of the lipid composition corresponded to the receptor. Given a total volume of 1.0 mL, this results in a receptor concentration of 0.1 mmol/L (1 equivalent). Based on a statistical distribution between the inner and outer leaflets of the lipid bilayer, the effective concentration of accessible receptors is estimated to be 0.05 mmol/L (0.5 equivalent, 50 nmol in 1.0 mL).

The interaction between AIE receptors and messengers is theoretically expected to follow stoichiometric ratios of 3:1 for H_3TREN^{3+} and 2:1 for H_2DET^{2+} and H_2EDA^{2+}.

The required ligand amount:

- **For a bidentate ligand** (binding 2 receptors): 0.5 receptor equivalents correspond to 0.25 (25 nmol) ligand equivalents.
- **For a tridentate ligand** (binding 3 receptors): 0.5 receptor equivalents correspond to 0.167 (16.67 nmol) ligand equivalents.

However, our experimental data indicate that receptor saturation often exceeds these theoretical ratios, particularly when signal transduction experiments were conducted in buffered solutions.

6. Influence of receptor concentration, lipid composition, and aqueous environment on AIEE-mediated signal transduction – supplementary data

Fluorescence emission was detected in all experiments even before the external addition of messenger molecules. At a receptor doping level of 1.25 mol%, no significant fluorescence changes were observed upon messenger titration (spectrum not shown), indicating that a minimal receptor density is required for effective signal transduction.

Increasing the receptor content to 2.5 mol% enabled the first detectable fluorescence enhancements. A further increase to 5 mol% led to significantly improved signal transduction, achieving stronger fluorescence emission with reduced messenger consumption. Liposomes with 10 mol% receptor exhibited a comparable fluorescence response to those with 5 mol%, but required fewer messenger equivalents to reach maximum signal intensity. Despite the doubled receptor concentration, no further increase in fluorescence was observed, suggesting that 5 mol% represents an optimal balance between receptor density and messenger efficiency.

The general affinity of the messengers for signal transduction was determined in the following order: $H_3TREN^{3+} \geq H_2DET^{2+} > H_2EDA^{2+}$.

A general assessment of messenger performance suggests that H_3TREN^3 efficiently induces fluorescence signals at minimal ligand concentrations, underscoring its high signal transduction efficiency. Notably, significant fluorescence increases were already detectable at 0.03 eq, whereas H_2DET^{2+} generally required higher concentrations to elicit measurable changes. If H_2DET^{2+} ultimately produces slightly higher fluorescence intensities than H_3TREN^3, this effect is accompanied by

substantially greater ligand consumption. H_2EDA^{2+} exhibited a response behavior comparable to H_2DET^{2+} but resulted in lower overall fluorescence intensities, making it the least effective messenger. These findings suggest that while H_2DET^{2+} maximizes signal intensity, H_3TREN^3 enables more efficient fluorescence generation at significantly lower ligand equivalents.

Figures S7–S15 illustrate fluorescence intensity changes depending on receptor concentration and aqueous environment. Figure S16 demonstrates the controlled down- and upregulation of the AIEE effect through dynamic modulation by Cu^{2+} and EDTA.

Note: In some cases, the apparent abrupt fluorescence increases stem from the consolidation of fluorescence spectra recorded at different stages of incremental messenger addition. Instead of displaying each individual titration step separately, cumulative effects were presented to maintain clarity, which may give the impression of a sudden fluorescence surge, although it actually results from successive messenger additions.

In other cases, significant fluorescence changes could only be induced by the addition of more concentrated messenger solutions, particularly in the presence of interfering buffer ions.

6.1 AIEE experiments in liposomes with distinct AIE receptor doping levels (2.5, 5, and 10mol%)—additional data

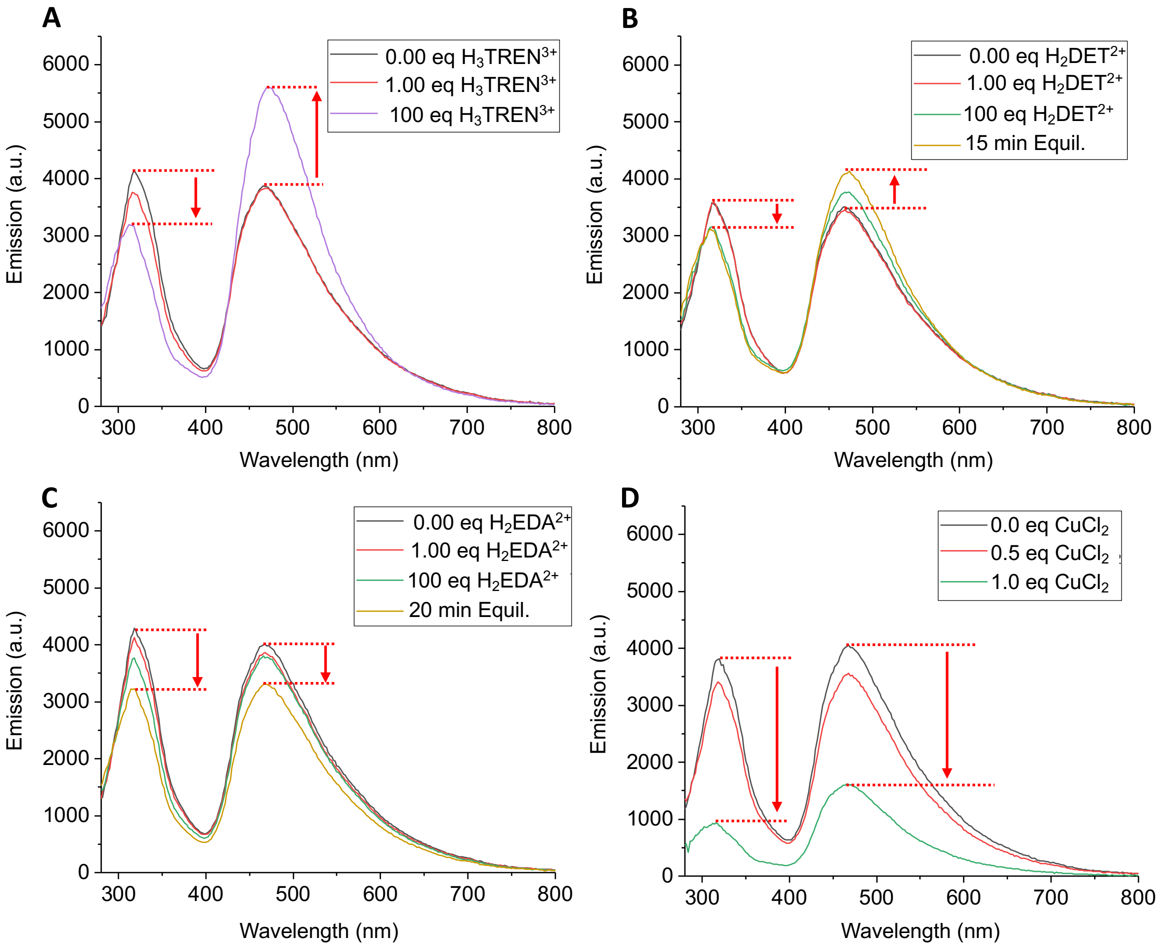

Fig. S7: Signal transduction experiments in phase-separated LUVs composed of the lipids DOPC/DPPC/Chol (1:2:1, c = 2 mM). The liposomes were doped with 2.5 mol% (0.05 mM) of receptor **1** and were suspended as an aqueous vesicle dispersion in MES buffer (375 mM, pH 5.5). Each experiment was performed by titrating a 100 µL sample with an external signaling molecule until fluorescence emission reached a stable level. Titration was performed using aqueous solutions of H_3TREN^{3+}, H_2DET^{2+}, H_2EDA^{2+} and $CuCl_2$. All fluorescence spectra exhibit two emission bands at ~312 nm and ~472 nm upon excitation at 230 nm, attributed to the bisphosphonate head group and the terminal AIE luminophore, respectively. A decrease in the bisphosphonate emission bands by 30% and 15% was observed. Fluorescence response at 472 nm of receptor **1** (A–D) upon titration, showing signal enhancements from 1.2-fold (B) to 1.5-fold (A), while titration with H_2EDA^{2+} and $CuCl_2$ (C–D) resulted in fluorescence decreases. Fluorescence enhancement at ~472 nm indicates messenger-induced receptor aggregation within the liposomes, while the concurrent decrease in emission at ~312 nm suggests complex formation between the messengers and the bisphosphonate receptor units. In contrast, the absence of a fluorescence increase upon H_2EDA^{2+} addition implies a preferential formation of 1:1 receptor-messenger complexes rather than receptor crosslinking. Notably, the fluorescence decrease at ~472 nm upon H_2EDA^{2+} titration suggests a messenger-induced receptor dispersion rather than clustering. This hypothesis is further supported by control experiments with non-crosslinking messengers, such as Cu^{2+} and guanidinium, which induced fluorescence quenching. The effect of guanidinium, comparable to that of H_2EDA^{2+}, is not included in the spectra presented.

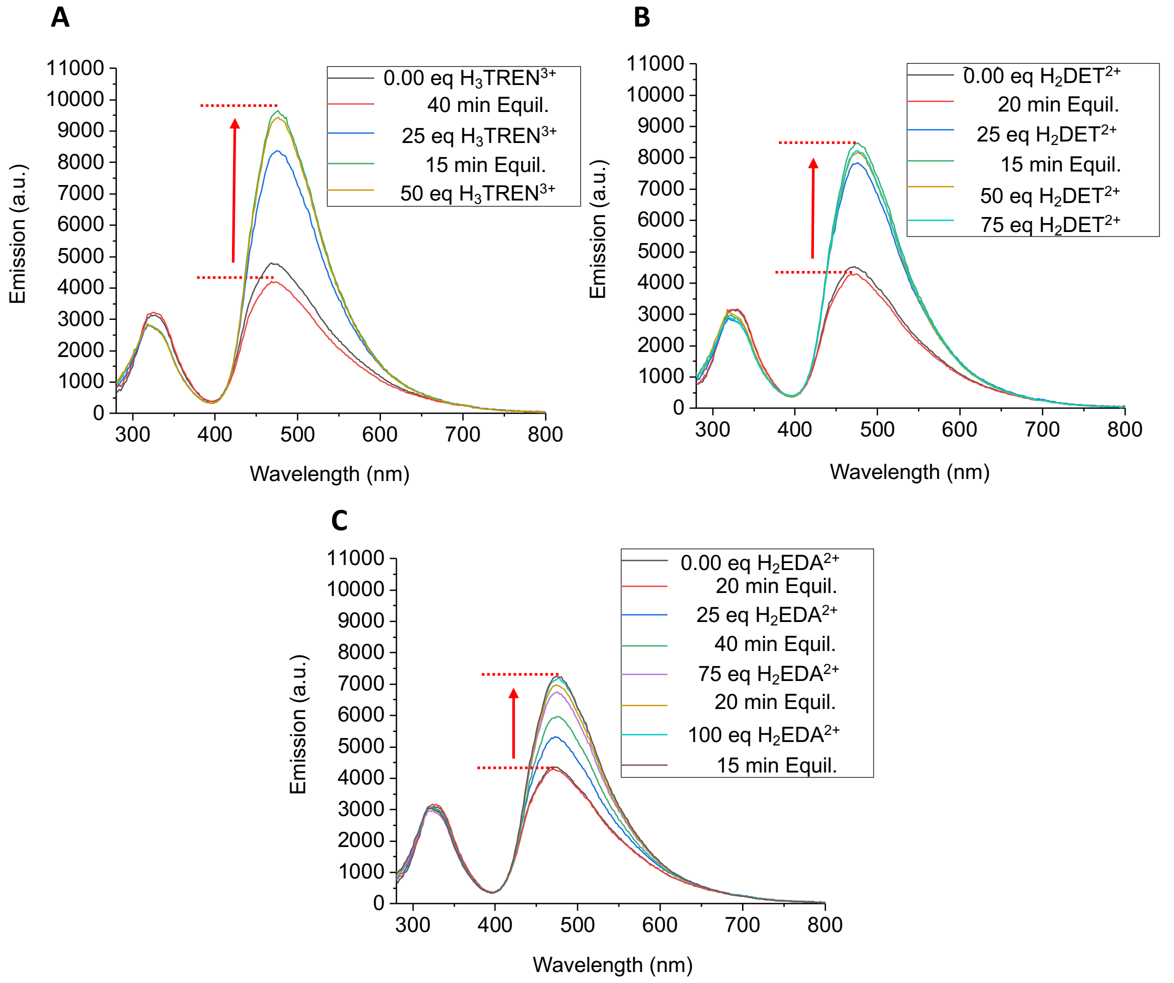

Fig. S8: Supplementary signal transduction experiments in phase-separated LUVs. The liposomes were doped with 5 mol% (0.1 mM) of receptor **1**. Experimental conditions and titration procedures were performed analogously to previous measurements, which are depicted in Fig. S 7. The vesicles exhibited a significantly improved response to messenger addition compared to initial experiments with 2.5 mol% receptor content. Increasing the receptor concentration to 5 mol% enhanced signal transduction efficiency, allowing for stronger fluorescence emission with reduced messenger consumption. Fluorescence response at 472 nm of receptor **1** (A–C) upon titration, showing signal enhancements from 1.7-fold (C) to 2.3-fold (A).

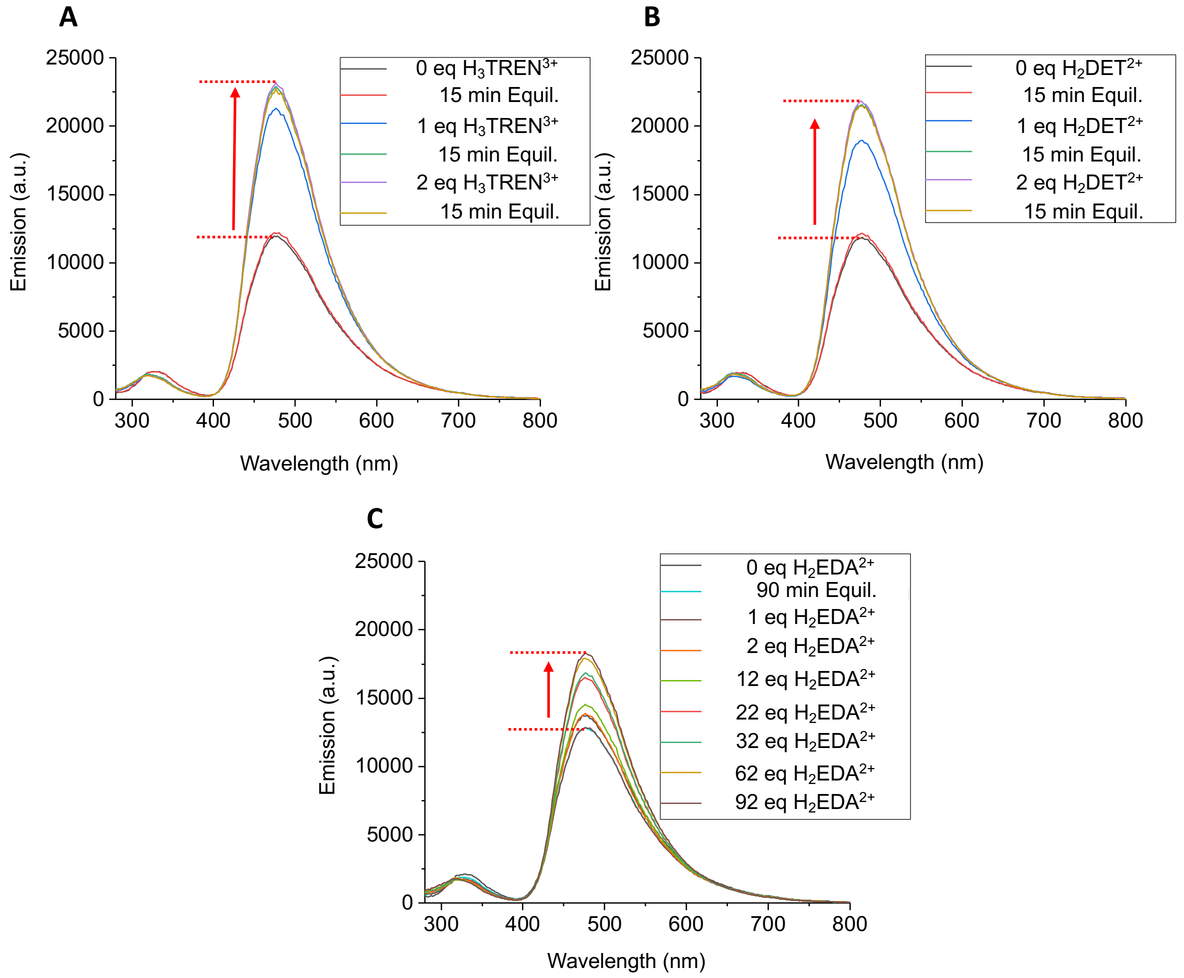

Fig. S9: Supplementary signal transduction experiments in phase-separated LUVs. The liposomes were doped with 10 mol% (0.2 mM) of receptor **1**. Experimental conditions and titration procedures were performed analogously to previous measurements, which are depicted in Fig. S 7. Liposomes with 10 mol% receptor exhibited a similar fluorescence response to those with 5 mol%, but required significantly less messenger to reach maximum signal intensity. Despite the doubled receptor concentration, no further increase in fluorescence emission was observed, indicating that 5 mol% receptor content is more efficient in terms of signal transduction relative to messenger consumption. Fluorescence response at 472 nm of receptor **1** (A–C) upon titration, showing signal enhancements from 1.4-fold (C) to 1.9-fold (A).

6.2 Messenger-induced signal transduction with AIE receptors in MES buffer—additional data

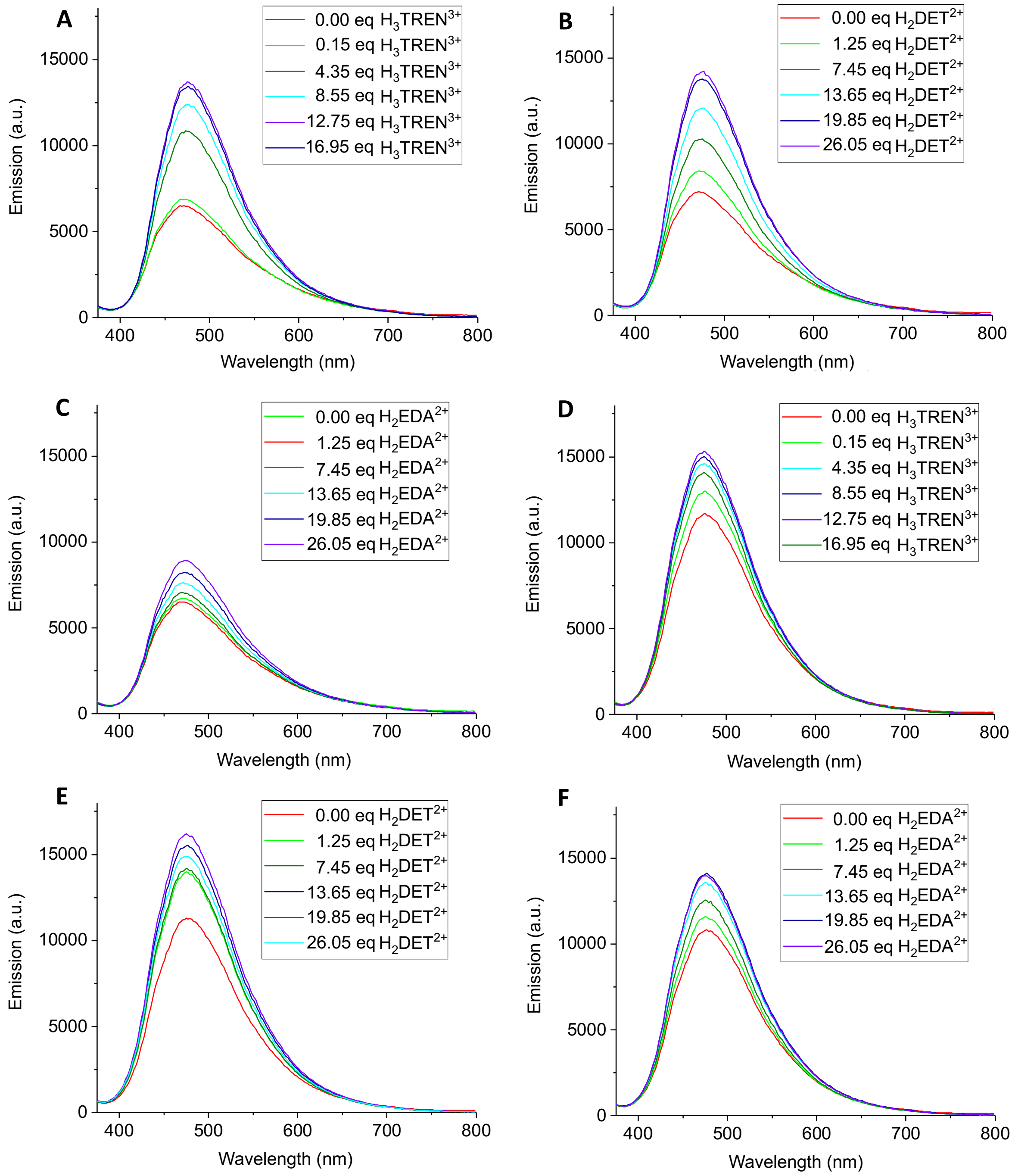

Fig. S10: Signal transduction experiments in phase-separated LUVs composed of the lipids DOPC/DPPC/Chol (1:2:1, c = 2 mM). The liposomes were doped with 5 mol% (0.1 mM) of receptor **1** or **2** and were suspended as an aqueous vesicle dispersion in MES buffer (375 mM, pH 5.5). Each experiment was performed by titrating a 60 µL sample with an external signaling molecule until fluorescence emission reached a stable level. Initially, titration was performed using aqueous solutions of H_3TREN^{3+} (V =2.15 µL, c = 93 µM, 0.03 eq), H_2DET^{2+} (V =1 µL, c = 1.5·mM, 0.25 eq) and H_2EDA^{2+} (V =0.75 µL, c = 2·mM, 0.25 eq). A more pronounced fluorescence increase was observed only with higher messenger concentrations: H_3TREN^{3+} (V = 2.7 µL, c = 9.3 mM, 4.2 eq), H_2DET^{2+} (V = 4.96 µL, c = 7.5 mM, 6.2 eq), and H_2EDA^{2+} (V = 1.86 µL, c = 20 mM, 6.2 eq). All titration experiments were conducted at 25 °C. Fluorescence response of receptor **1** (A–C) upon titration, with signal enhancements ranging from 1.4-fold (C) to 2.1-fold (A), while receptor **2** (D–F) exhibited increases between 1.3-fold and 1.4-fold (D-F).

6.3 Messenger-induced signal transduction with AIE receptors in HEPES buffer—additional data

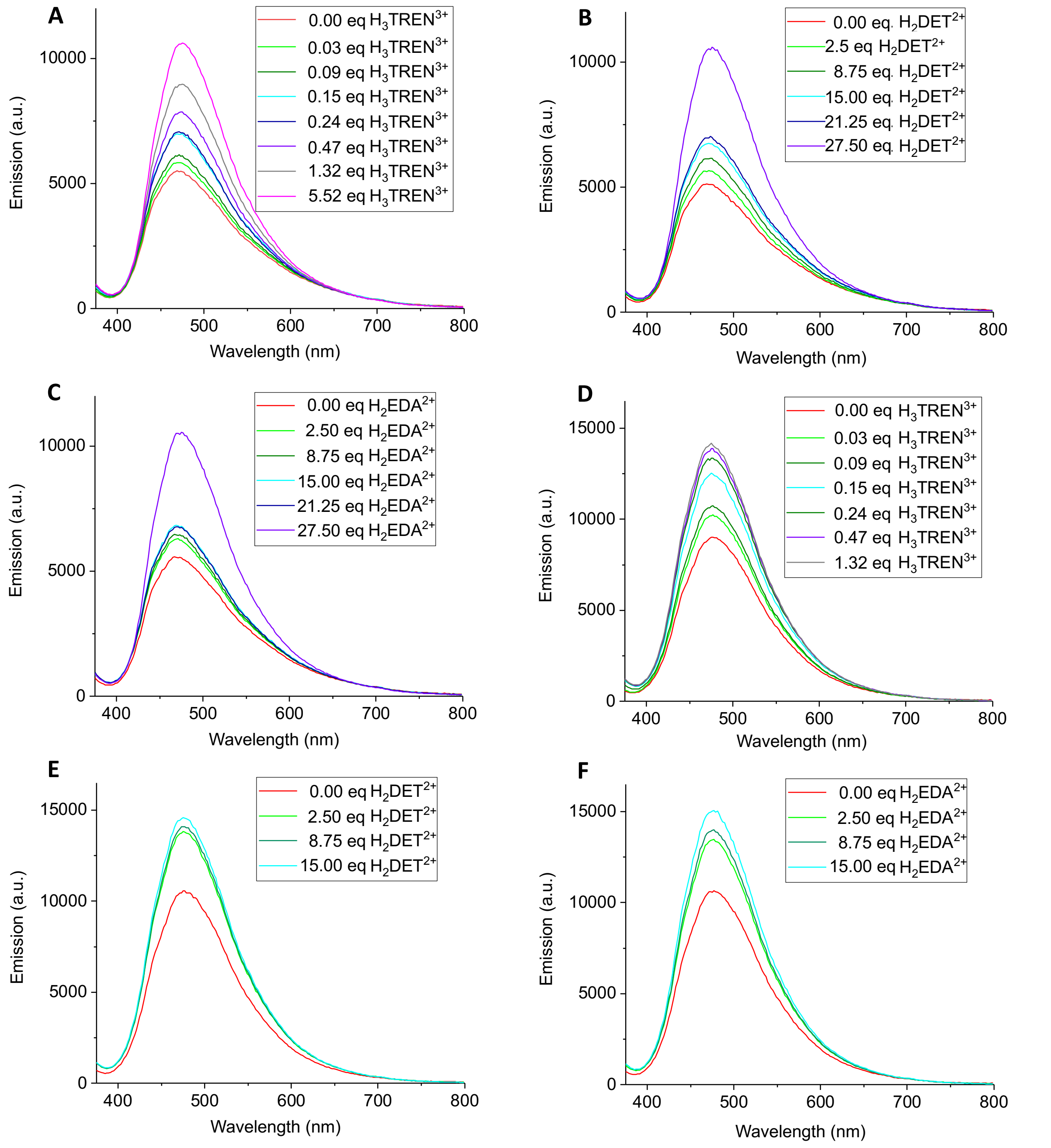

Fig. S11: Signal transduction experiments in phase-separated LUVs composed of the lipids DOPC/DPPC/Chol (1:2:1, c = 2 mM). The liposomes were doped with 5 mol% (0.1 mM) of receptor **1** or **2** and were suspended as an aqueous vesicle dispersion in HEPES buffer (100 mM, pH 7.4). Each experiment was performed by titrating a 60 µL sample with an external signaling molecule until fluorescence emission reached a stable level. The titration was performed using aqueous solutions of H_3TREN^{3+} (1. V =2.15 µL, c = 93 µM, 0.03 eq; 2. V = 1.08 µL, c = 0.93 mM, 0.17 eq; V = 2.7 µL, c = 9.3 mM, 4.2 eq), H_2DET^{2+} (1. V =1 µL, c = 1.5 mM, 0.25 eq; 2. V =2.48 µL, c = 15.1 mM, 6.2 eq) and H_2EDA^{2+} (1. V =0.75 µL, c = 2 mM, 0.25 eq; 2. V = 1.86 µL, c = 20·mM, 6.2 eq). All titration experiments were conducted at 25 °C. Fluorescence response of receptor **1** (A–C) upon titration, with signal enhancements ranging from 1.9-fold (A and C) to 2.1-fold (B), while receptor **2** (D–F) exhibited increases between 1.4-fold (E and F) and 1.6-fold (D).

6.4 Transmembrane signaling experiments with AIE receptors in heterogeneous vesicles with Ld/Lo phase coexistence in NaCl solution—additional data

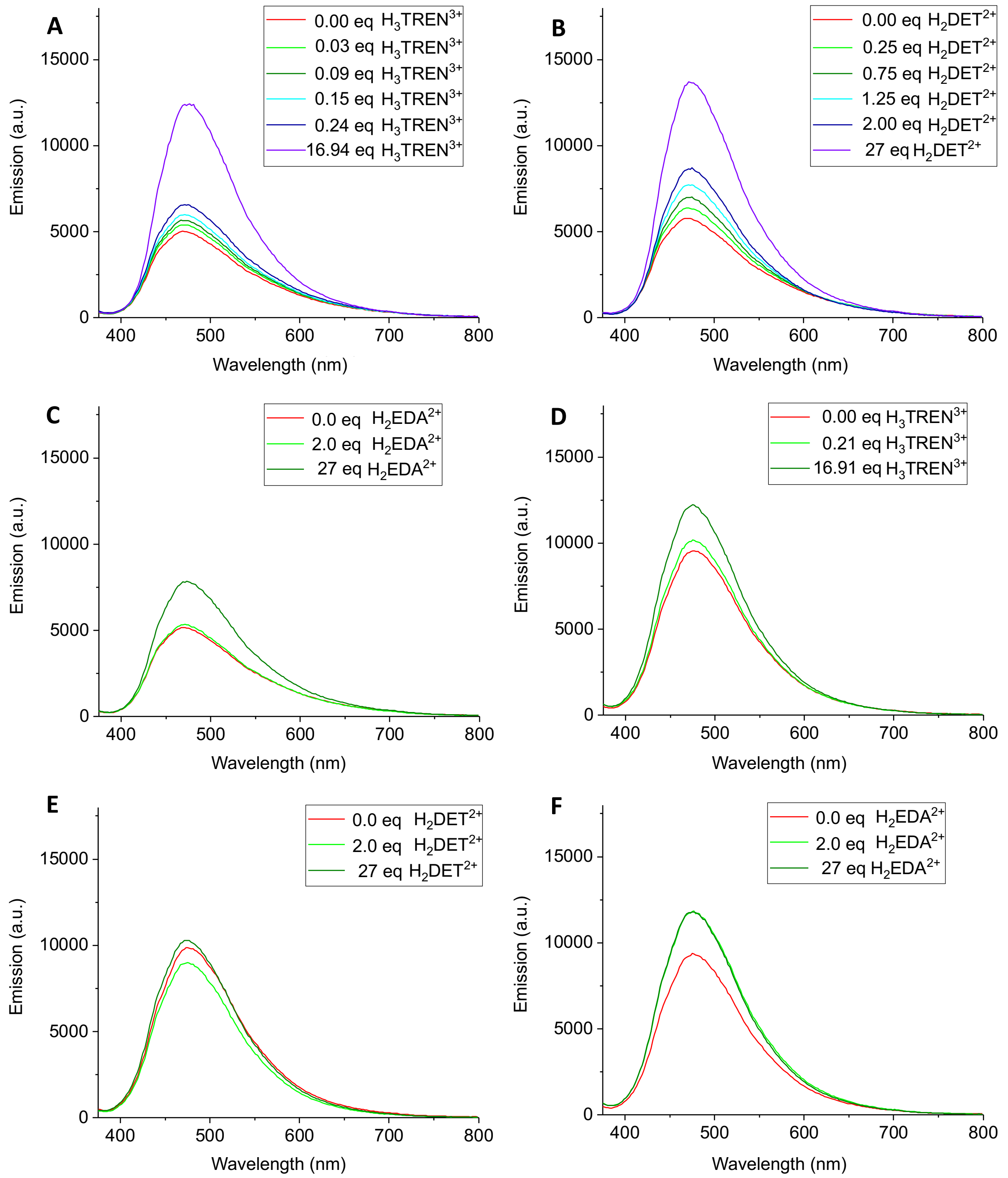

Fig. S12: Signal transduction experiments in phase-separated LUVs composed of the lipids DOPC/DPPC/Chol (1:2:1, c = 2 mM). The liposomes were doped with 5 mol% (0.1 mM) of receptor **1** or **2** and suspended in NaCl solution (100 mM, pH 7.4). Each experiment was performed by titrating a 60 µL sample with an external signaling molecule until fluorescence emission reached a stable level. The titration was performed using aqueous solutions of H_3TREN^{3+} (V =2.15 µL, c = 93 µM, 0.03 eq), H_2DET^{2+} (V =1 µL, c = 1.5 mM, 0.25 eq) and H_2EDA^{2+} (1. V =0.75 µL, c = 2 mM, 0.25 eq; 2. V = 7.5 µL, c = 20 mM, 25 eq). Initial minor fluorescence increases were observed, followed by slight intensity decreases. For H_2EDA^{2+}, the fluorescence increase was negligible at lower concentrations, while a minor signal enhancement was observed with higher-concentration messenger solution. For spectra A and B, a more pronounced fluorescence increase was only observed at higher messenger concentrations: H_3TREN^{3+} (V = 10.8 µL, c = 9.3 mM, 16.7 eq) and H_2DET^{2+} (V = 9.9 µL, c = 15.1 mM, 25 eq). All titration experiments were conducted at 25 °C. Fluorescence response of receptor **1** (A–C) upon titration, with signal enhancements ranging from 1.5-fold (C) to 2.5-fold (B), while receptor **2** (D–F) exhibited only weak fluorescence, with a maximum increase of approximately 1.4-fold.

6.5 Signal transduction experiments in LUVs with a purely Ld-phase membrane composed of DOPC in pure water (pH 7.4) —additional data

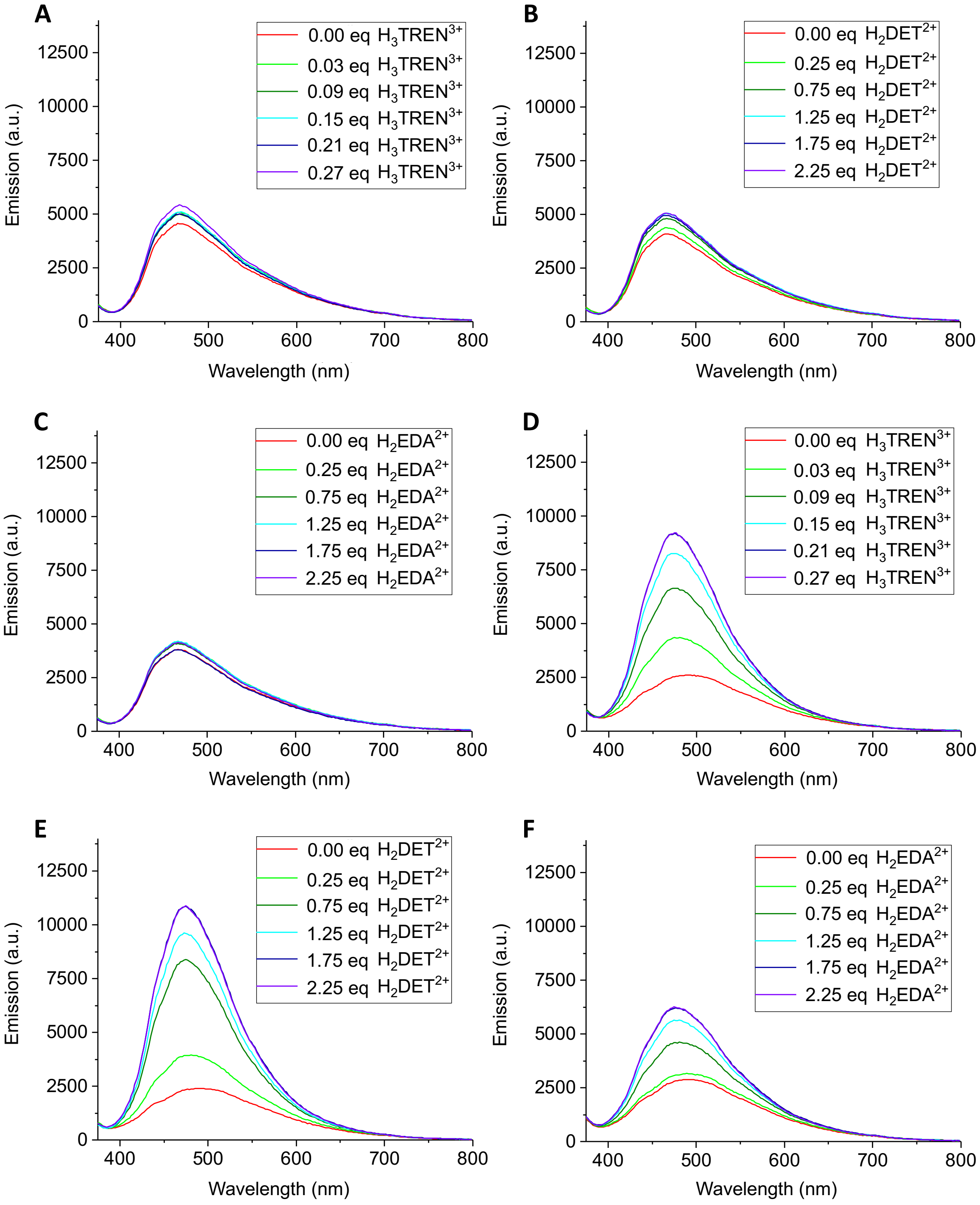

Fig. S13: Signal transduction experiments in LUVs with a purely Ld-phase membrane composed of DOPC (c = 2 mM). The liposomes were doped with 5 mol% (0.1 mM) of receptor **1** or **2** and suspended in ultrapure water (pH 7.4, adjusted with NaOH) to minimize interference from competing ions (e.g., from buffer components) while maintaining physiological pH conditions. Each experiment was performed by titrating a 60 µL sample with an external signaling molecule until fluorescence emission reached a stable level. The titration was performed using aqueous solutions of H_3TREN^{3+} (V =2.15 µL, c = 93 µM, 0.03 eq), H_2DET^{2+} (V =1 µL, c = 1.5 mM, 0.25 eq) and H_2EDA^{2+} (V =0.75 µL, c = 2·mM, 0.25 eq). All titration experiments were conducted at 25 °C. Upon titration, receptor **1** (A–C) did not exhibit a significant AIEE effect with the applied messengers, while receptor **2** (D–F) exhibited fluorescence enhancements ranging from 2.2-fold (F) to 4.5-fold (E), with an intermediate increase of 3.5-fold (D). The observed fluorescence response highlights that the membrane environment can differentially affect structurally related yet distinct receptors.

6.6 Transmembrane signaling experiments with AIE receptors in heterogeneous vesicles with Ld/Lo phase coexistence performed in ultrapure water – additional data

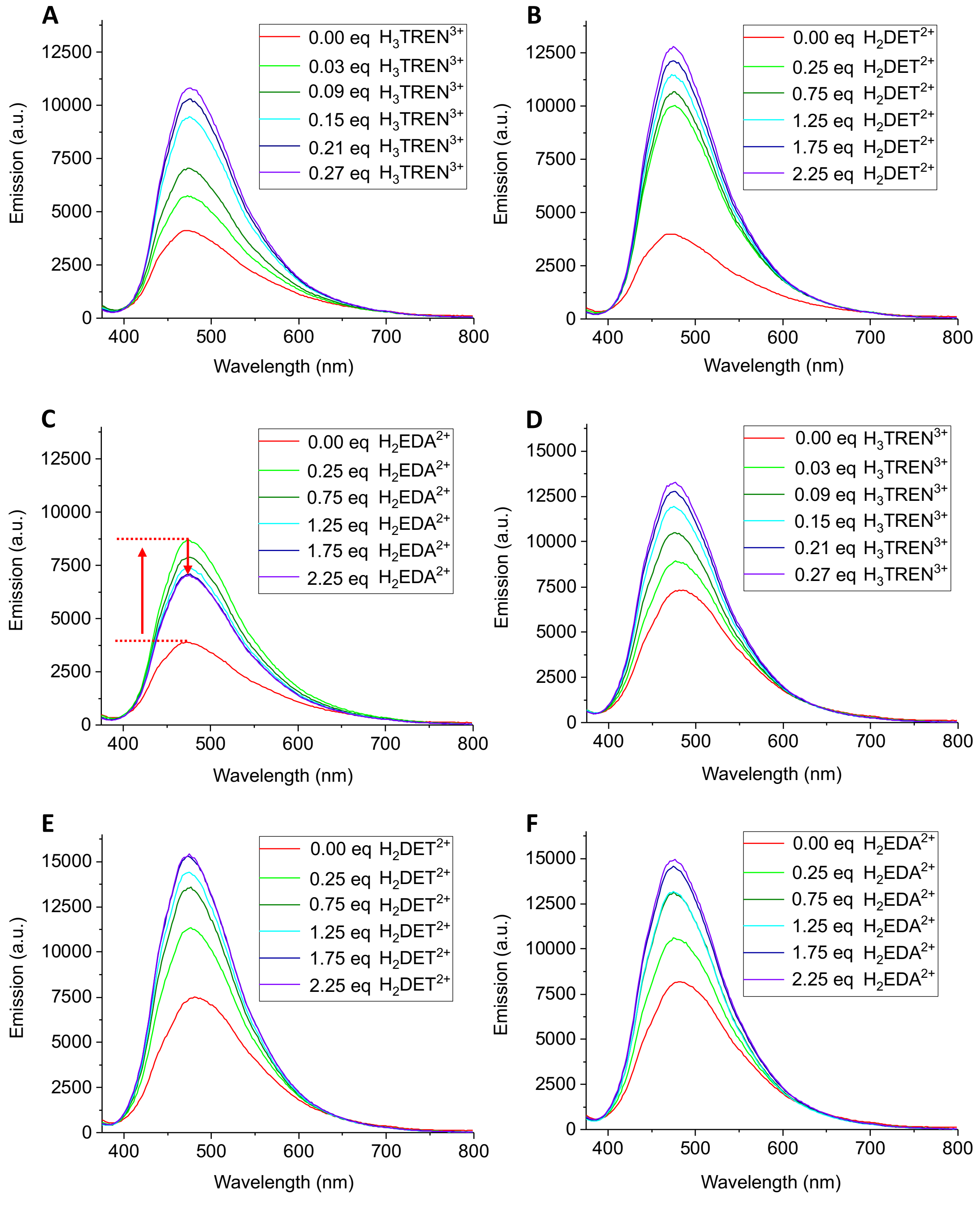

Fig. S14: Signal transduction experiments in phase-separated LUVs composed of the lipids DOPC/DPPC/Chol (1:2:1, c = 2 mM). The liposomes were doped with 5 mol% (0.1 mM) of receptor **1** or **2** and suspended in ultrapure water (pH 7.4, adjusted with NaOH) to minimize interference from competing ions (e.g., from buffer components) while maintaining physiological pH conditions. Each experiment was performed by titrating a 60 µL sample with an external signaling molecule until fluorescence emission reached a stable level. The titration was performed using aqueous solutions of H_3TREN^{3+} (V =2.15 µL, c = 93 µM, 0.03 eq), H_2DET^{2+} (V =1 µL, c = 1.5·mM, 0.25 eq) and H_2EDA^{2+} (V =0.75 µL, c = 2·mM, 0.25 eq). All titration experiments were conducted at 25 °C. Fluorescence response of receptor **1** (A–C) upon titration, with signal enhancements ranging from 2.6-fold (A) to 3.2-fold (B), while receptor **2** (D–F) exhibited increases between 1.8-fold (D and F) and 2-fold (E). The induced fluorescence signals remained relatively stable and continuous throughout and beyond the titration process, indicating reproducible fluorescence responses, except for spectrum C, where a slight decrease was observed.

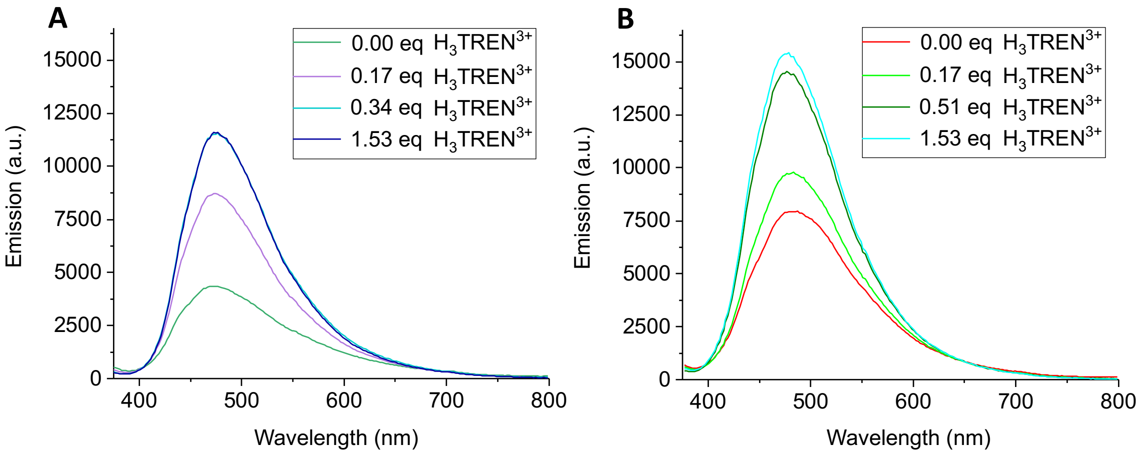

Fig. S15: Supplementary experiment to Figure 3: Fluorescence response of receptors **1** (A) and **2** (B) upon titration with a higher concentration of H_3TREN^{3+} (V = 1.1 µL, c = 0.93 mM, 0.17 eq) for improved comparability with H_2DET^{2+}, to assess whether an increased H_3TREN^{3+} concentration leads to a further fluorescence enhancement, as previously observed for H_2DET^{2+} in Fig. S10. The titration was performed until fluorescence emission reached a stable level, with no further detectable changes. Despite the titration with a higher messenger concentration, no further fluorescence enhancement was observed, yielding results comparable to previous experiments.

7. Kinetic analysis within a typical titration experiment

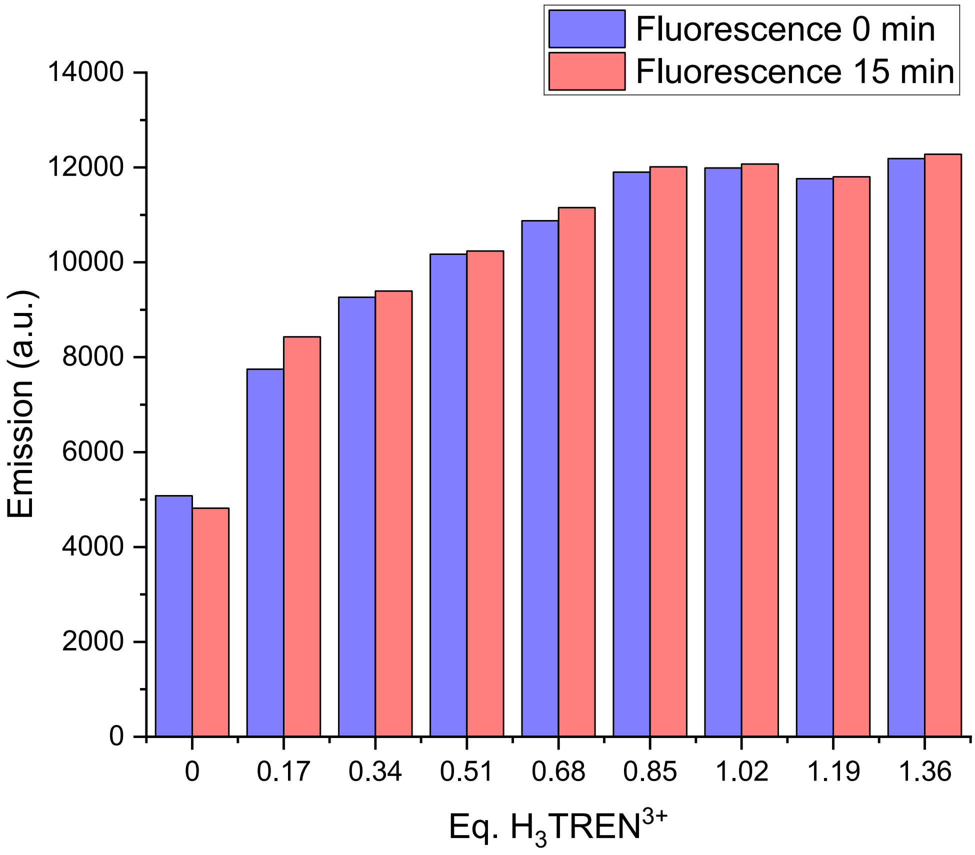

Fig. S16: Titration of receptor **1** in a DOPC/DPPC/Chol vesicle with aliquots of messenger H_3TREN^{3+}. The column pairs show an immediate fluorescence increase at t = 0, which remains largely unaltered after 15 min. Note the substoichiometric and nonlinear effect related to receptor clustering within the vesicle membrane.

8. Background to signal ratio in a typical signaling experiment

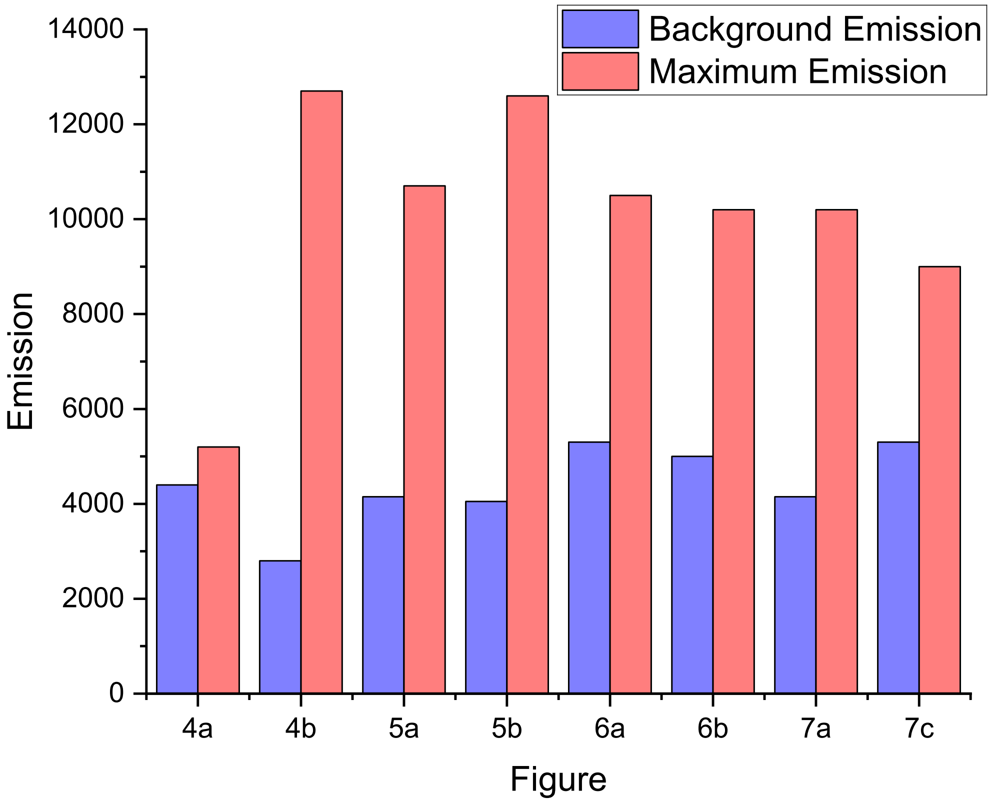

Fig. S17: Calculation of background fluorescence relative to total induced fluorescence (signal) for signaling experiments in Figs. 4-7. Note background suppression in experiment 4b relating to the unfavorable homogeneous DOPC lipid environment.

9. References

[1] C. A. Ilioudis, K. S. Hancock, D. G. Georganopoulou and J. W. Steed, *New Journal of Chemistry* **2000**, *24*, 787-798.

[2] a) D. M. Rock and R. L. Macdonald, *Annual review of pharmacology and toxicology* **1995**, *35*, 463-482; b) J. W. Steed and J. L. Atwood, *Supramolecular chemistry*, John Wiley & Sons, **2013**, p.

[3] A. G. Blackman, *Polyhedron* **2005**, *24*, 1-39

[4] K. Bernitzki, M. Maue and T. Schrader, *Chem. Eur. J.* **2012**, *18*, 13412-13417.

[5] A. Bencini, A. Bianchi, E. Garcia-Espana, M. Micheloni and J. A. Ramirez, *Coordination chemistry reviews* **1999**, *188*, 97-156.

[6] a) K. Klein, M. Hayduk, S. Kollenda, M. Schmiedtchen, J. Voskuhl and M. Epple, *Molecules* **2022**, *27*, 1788; b) J. S. Ishibashi, J. L. Marshall, A. Maziere, G. J. Lovinger, B. Li, L. N. Zakharov, A. Dargelos, A. Graciaa, A. Chrostowska and S.-Y. Liu, *Journal of the American Chemical Society* **2014**, *136*, 15414-15421.
[7] L. Rojas-Sánchez, V. Sokolova, S. Riebe, J. Voskuhl and M. Epple, *ChemNanoMat* **2019**, *5*, 436-446.

[8] S. Riebe, A. Zimmermann, J. Koch, C. Vallet, S. K. Knauer, A. Sowa, C. Wölper and J. Voskuhl, *RSC advances* **2020**, *10*, 19643-19647.

[9] M. Maue, K. Bernitzki, M. Ellermann and T. Schrader, *Synthesis* **2008**, *2008*, 2247-2256.

[10] a) G. M. Salamończyk, *Tetrahedron letters* **2011**, *52*, 155-158; b) M. Engel, C. W. Burris, C. A. Slate and B. W. Erickson, *Tetrahedron* **1993**, *49*, 8761-8770.

[11] H. Choi, H. J. Shirley, P. A. Hume, M. A. Brimble and D. P. Furkert, Angewandte Chemie 2017, 129, 7528-7532.

[12] a) P. V. Torchilin, V. Torchilin, V. Torchilin and V. Weissig, *Liposomes: a practical approach*, Oxford University Press, **2003**; b) H. Zhang, *Liposomes: Methods and protocols* **2017**, 17-22.

[13] P. Uppamoochikkal, S. Tristram-Nagle and J. F. Nagle, *Langmuir* **2010**, *26*, 17363-17368.

[14] M. Hope, M. Bally, L. Mayer, A. Janoff and P. Cullis, *Chemistry and physics of lipids* **1986**, *40*, 89-107.
